# Detecting Potentially Harmful and Protective Suicide-Related Content on Twitter: Machine Learning Approach

**DOI:** 10.2196/34705

**Published:** 2022-08-17

**Authors:** Hannah Metzler, Hubert Baginski, Thomas Niederkrotenthaler, David Garcia

**Affiliations:** 1 Section for the Science of Complex Systems Center for Medical Statistics, Informatics and Intelligent Systems Medical University of Vienna Vienna Austria; 2 Unit Suicide Research and Mental Health Promotion Center for Public Health Medical University of Vienna Vienna Austria; 3 Complexity Science Hub Vienna Vienna Austria; 4 Computational Social Science Lab Institute of Interactive Systems and Data Science Graz University of Technology Graz Austria; 5 Institute for Globally Distributed Open Research and Education Vienna Austria; 6 Institute of Information Systems Engineering Vienna University of Technology Vienna Austria

**Keywords:** suicide prevention, Twitter, social media, machine learning, deep learning

## Abstract

**Background:**

Research has repeatedly shown that exposure to suicide-related news media content is associated with suicide rates, with some content characteristics likely having harmful and others potentially protective effects. Although good evidence exists for a few selected characteristics, systematic and large-scale investigations are lacking. Moreover, the growing importance of social media, particularly among young adults, calls for studies on the effects of the content posted on these platforms.

**Objective:**

This study applies natural language processing and machine learning methods to classify large quantities of social media data according to characteristics identified as potentially harmful or beneficial in media effects research on suicide and prevention.

**Methods:**

We manually labeled 3202 English tweets using a novel annotation scheme that classifies suicide-related tweets into 12 categories. Based on these categories, we trained a benchmark of machine learning models for a multiclass and a binary classification task. As models, we included a majority classifier, an approach based on word frequency (term frequency-inverse document frequency with a linear support vector machine) and 2 state-of-the-art deep learning models (Bidirectional Encoder Representations from Transformers [BERT] and XLNet). The first task classified posts into 6 main content categories, which are particularly relevant for suicide prevention based on previous evidence. These included personal stories of either suicidal ideation and attempts or coping and recovery, calls for action intending to spread either problem awareness or prevention-related information, reporting of suicide cases, and other tweets irrelevant to these 5 categories. The second classification task was binary and separated posts in the 11 categories referring to actual suicide from posts in the off-topic category, which use suicide-related terms in another meaning or context.

**Results:**

In both tasks, the performance of the 2 deep learning models was very similar and better than that of the majority or the word frequency classifier. BERT and XLNet reached accuracy scores above 73% on average across the 6 main categories in the test set and *F*_1_-scores between 0.69 and 0.85 for all but the suicidal ideation and attempts category (*F*_1_=0.55). In the binary classification task, they correctly labeled around 88% of the tweets as about suicide versus off-topic, with BERT achieving *F*_1_-scores of 0.93 and 0.74, respectively. These classification performances were similar to human performance in most cases and were comparable with state-of-the-art models on similar tasks.

**Conclusions:**

The achieved performance scores highlight machine learning as a useful tool for media effects research on suicide. The clear advantage of BERT and XLNet suggests that there is crucial information about meaning in the context of words beyond mere word frequencies in tweets about suicide. By making data labeling more efficient, this work has enabled large-scale investigations on harmful and protective associations of social media content with suicide rates and help-seeking behavior.

## Introduction

### Background

Suicide is a major public health problem worldwide, accounting for 1.4% of all deaths, equaling almost 800,000 in 2017, with many more suicide attempts [1]. Research shows that exposure to suicide-related news media content can influence suicidal behavior in vulnerable individuals in both harmful and beneficial ways. Whether suicide cases increase or decrease after exposure to suicide-related news seems to depend on specific elements of media content and language. As a recent meta-analysis of media effects research on suicide shows, most solid evidence exists for increases in suicides after exposure to news about celebrity deaths by suicide [2]. This imitation of suicidal behavior is commonly referred to as the Werther effect [3]. In contrast, exposure to other types of content may have a protective effect, with the strongest evidence existing for stories of hope, recovery, and coping [4-6]. However, broader prevention texts (ie, texts focused on prevention that were not personal stories of recovery) have also been found to be associated with protective effects in some studies [7,8]. The association of positive messaging on suicide prevention with later decreases in suicide rates has been labeled as the Papageno effect [4].

Studies investigating how exposure to media content is associated with suicidal behavior have mainly focused on traditional news outlets, such as print and web-based newspapers, radio, and television broadcasts. Investigations of the associations between social media content and suicides remain extremely scarce [9-13]. Most of the previous research on social media has focused on detecting suicidal ideation in users’ posts with the purpose to identify individuals at risk, but very little research has been conducted to analyze media effects. The applied methods for identifying such individuals include machine learning as well as word dictionaries, word frequencies, topic models, and social network analysis (eg, [14-18]; for more information, see reviews by Bernert et al [19], Castillo-Sánchez G [20], Ji et al [21], Wongkoblap et al [22], and Yin et al [23]). A small number of studies have started developing machine learning classifiers for content other than suicidal ideation, despite evidence from research on traditional media that other content types can affect suicidal behavior (eg, [2,4]).

### Limitations of Previous Similar Machine Learning Studies

A machine learning study categorized tweets according to expressed emotions [9], whereas 2 further studies [24,25] classified typically occurring content types, including celebrity suicide reports, suicidal intent, awareness campaigns, prevention information, condolences, and flippant remarks. Although these 2 studies include several different prevention-relevant content types, they both use the same and relatively small data set, which is limited to tweets containing celebrity names or suicidal intent. Furthermore, all these machine learning studies have used word frequency statistics as predefined features for model training, which cannot capture differences in the meaning of words across different contexts. This study addresses several gaps in the existing literature on media effects on suicidal behavior. The first is the lack of research on suicide-related social media content other than suicidal ideation. Suicide is a leading cause of deaths among young adults [1] who predominantly receive news on such platforms [13,26]. This highlights the urgency of systematic research on social media effects. In addition, social media posts often feature other content types than traditional news outlets, on which research is required. This includes diary-like posts in which people describe their personal experiences or posts addressed to their social network with the intention to prevent suicides. In this study, we investigated Twitter data and created a detailed annotation scheme for the types of suicide-related tweets that are potentially relevant to prevention efforts.

Second, regarding prevention-related media content, there is currently a discussion in the literature on whether content that highlights prevalence data to increase problem awareness has a protective effect or may even be detrimental [27]. By highlighting the prevalence of suicide and risk factors such as mental health or abuse without mentioning solutions to the issue, attempts to spread awareness may normalize suicidal behavior and trigger harmful effects [28]. In this study, we have addressed the lack of studies differentiating between prevention messages focusing on prevalence and prevention opportunities [27]. Specifically, we distinguish between awareness and prevention-focused calls for action on Twitter.

Third, the samples used in previous studies on suicide-related social media content are limited either in size (eg, [10,11]) or by a set of search terms used to collect tweets (eg, [12,29]). Sample sizes are usually small as all content needs to be annotated manually. Search terms either narrowly focus on events such as the suicide of specific celebrities (eg, [12]) or broadly include all texts containing the word suicide. Therefore, the effects of different content types may cancel each other out [2]. Thus, to systematically investigate the potentially harmful and protective effects, a large-scale and simultaneously fine-grained approach is necessary.

### Overview of This Study

We have addressed these challenges by first developing a comprehensive annotation scheme that systematically organizes tweets about suicide into categories most likely to beneficially or harmfully affect suicidal and help-seeking behavior based on available evidence (eg, [2,4,6,30]). Second, we compared different natural language processing and machine learning methods to automatically detect and classify particularly important categories in large quantities of social media data. Extending previous work on different prevention-related social media content types [9,24,25], we included not only word frequency–based models but also 2 deep learning models that can capture content-dependent meanings of words [16]. We trained all models in two tasks: a multiclass classification problem with 6 main content categories and a binary classification problem of tweets about actual suicide versus off-topic tweets, which use the word suicide in another meaning or context.

The 6 main categories assessed include 5 content types that are particularly relevant for suicide prevention based on previous research. As described earlier, the strongest evidence exists for celebrity suicide case reports having harmful effects and for personal stories of hope and coping having protective effects. A second type of personal stories in Twitter posts mentions suicidal ideation and attempts without any hint at coping or recovery. Preliminary evidence suggests that such posts may have a protective effect [11]. Some evidence also suggests protective effects for general prevention messages [7,8]. We have distinguished between general prevention messages calling for action by either spreading prevention-related information or solution-oriented attitudes from those spreading problem awareness only. Finally, we included an irrelevant category to identify tweets outside the other 5 possible categories described.

### Objectives

The objective of our study was to enable large-scale studies on the association between tweet content and suicidal and help-seeking behaviors. Specifically, we aimed to provide volume estimates for the different prevention-relevant tweet categories for follow-up studies on the associations of these estimates with the number of suicide cases and helpline calls.

## Methods

### Data Set for Training Machine Learning Models

Given that this study is part of a project on media messaging for suicide prevention in the United States, all data sets of this study include English tweets of users located in the United States. We retrieved tweet IDs via the data reseller Crimson Hexagon (now known as Brandwatch), previously used for suicide research [9,12], and then downloaded the full text of these tweets via the Twitter application programming interface. Crimson Hexagon provides access to the entire history of Twitter data and includes reliable language and location filters. The location algorithm matches 90% of all posts in a country to a location using a combination of geocoordinates, location information from user profiles, and users’ time zones and languages [1].

Using a list of keywords and exclusion terms, we created a pool of unique tweets without duplicates or retweets, based on which we prepared a labeled set of tweets for training the machine learning models. We retrieved tweets posted between January 1, 2013, and May 31, 2020 (see note on dates in Multimedia Appendix 1), which contained at least one of the suicide-related search terms taken from a previous study [11]. The search terms were *suicide*, *suicidal*, *killed himself*, *killed herself*, *kill himself*, *kill herself*, *hung himself*, *hung herself*, *took his life, took her life*, *take his life*, *take her life*, *end his own life*, *end her own life*, *ended his own life*, *ended her own life*, *end his life*, *end her life*, *ended his life*, *ended her life*, *ends his life*, and *ends her life*.

The exclusion terms were identified by inspecting word frequency plots for common terms that may indicate that tweets used the term suicide to describe something other than someone ending their life or terms that indicated tweets about suicide bombing. We then verified whether these terms were actual mismatches by reading examples of tweets containing these terms. Thus, tweets with the most common use of the term suicide in contexts that do not refer to actual suicide could be excluded. The final list of exclusion terms was *suicide squad (a movie)*, *suicidechrist*, SuicideGirl* (a website featuring pin-up photographs of models), SuicideBoy* (male models), *suicideleopard (a frequently mentioned Twitter user)*, suicidexjockey* (a Twitter user), *suicidal grind (a music album)*, *Epstein (excessive speculations about whether the death of Jeffrey Epstein was or was not a suicide)*, *political suicide (tweets using suicide as a metaphor for political failure)*, *Trump*, clinton*, *Hillary*, *Biden*, and *sanders (also mostly about political suicide)*.

To avoid overlearning from multiple identical tweets, we ensured that the labeled data used for machine learning did not include any tweet duplicates. We excluded retweets (tweets categorized as retweets by Crimson Hexagon given the metadata of tweets as well as tweets containing the manual labels RT for retweets or MT for slightly modified tweets). We assembled a labeled data set of 3202 tweets by iteratively selecting tweets from a larger pool of tweets as described in the *Creating the Annotation Scheme and Labeled Data Set* section. We refer to these 3202 tweets as the *total labeled data set.* Although part of this data set was combined using keywords and model predictions (see below), a second subsample of 1000 tweets was selected randomly. We refer to these 1000 tweets as the *randomly selected labeled data set.*

In the course of the study, we combined two other data sets: the first to compare model and human interrater reliability and the second for a face validity check and a follow-up study (Niederkrotenthaler et al, unpublished data, May 2022; see Multimedia Appendix 1 for details). Both are described in detail in the *Evaluating* *Reliability and Face Validity of Model Predictions for BERT* section.

### Creating the Annotation Scheme and Labeled Data Set

**Figure 1:**
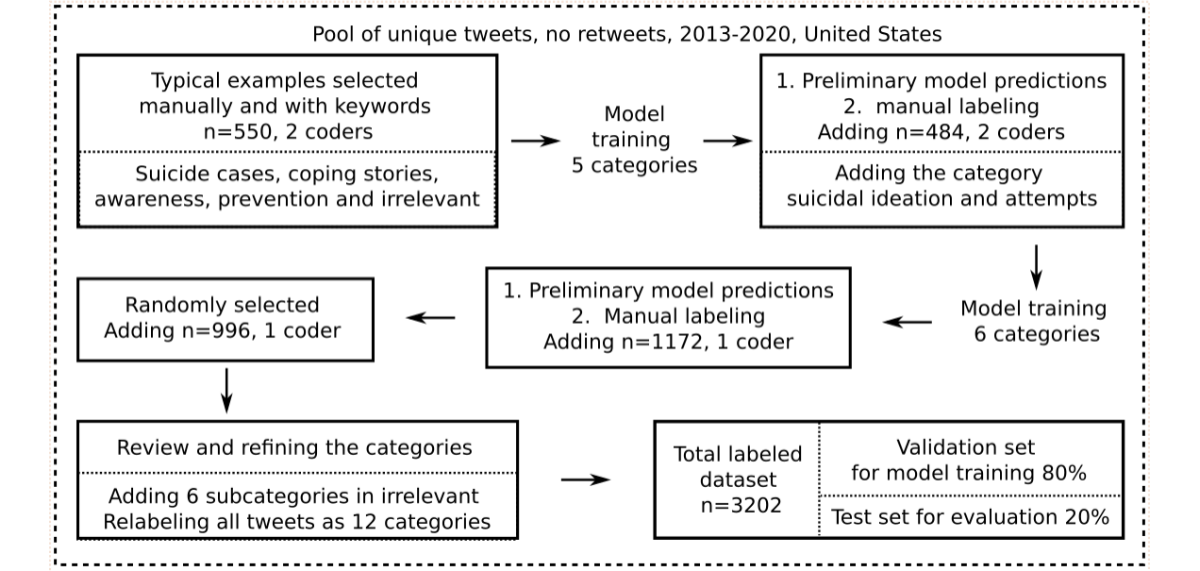
Creating the labeled data set and annotation scheme. Each box describes how tweets were selected from the large pool of available tweets, how many tweets were added to the training data set in each step (after removing duplicates), and how many coders labeled each tweet. When we used preliminary model predictions to identify potential candidates for each category, we deleted the model labels before manual coding. After rounds with 2 coders, we checked interrater reliability, adapted the annotation scheme until all disagreements were clarified, and relabeled the respective sample.

Creating the annotation scheme and the labeled data set was an iterative human-in-the-loop process building on preliminary classifiers and annotations. We started with 5 broad categories which appeared most relevant, given previous research on traditional media (see the *Introduction* section). We then added additional categories when tweets did not fit into the existing categories but might nevertheless be associated with suicides. Given that the tweets of interest are relatively rare compared with irrelevant tweets, we used the following stepwise procedure to identify examples, which is also illustrated in Figure 1.

1. We manually selected approximately 100 tweets for each of the 5 main categories (550 tweets in total): *suicide cases, coping stories, awareness, prevention,* and *irrelevant tweets.* To gather the first set of tweets, we searched the data set for typical examples, both randomly and with keywords that might indicate each particular category. We iteratively expanded the list of keywords by inspecting the most frequent terms in the resulting tweets in a systematic way [31]. The full list of keywords is provided in Multimedia Appendix 1; examples are *committed or found dead* for suicide cases, recover* or *hope* for coping stories, *lifeline or prevention* for prevention, *awareness,* and *please retweet* or *please copy* for awareness.
2. We used a preliminary machine learning model to make predictions based on the first training data set of 550 tweets to identify potential examples for each category. Next, two authors with domain expertise (TN and HM) continued annotating 100 tweets from each of the 5 predicted categories (484 after removing duplicates and missing labels from a coder). The interrater reliability for these 500 tweets was a Cohen κ of 0.75. On the basis of a careful inspection of all disagreements, we refined the definitions for all categories and adjusted the labels of all previously annotated tweets accordingly. Annotating these tweets, we further noticed a novel type of message not described in research on traditional news reporting, namely purely negative descriptions of suicidal experiences without any hint at coping, hope, or recovery. We updated the annotation scheme to include this new category *suicidal ideation and attempts*, resulting in 6 main categories. The total training set of tweets included 1034 tweets.
3. At this stage of the labeling process, we found that two dimensions were generally helpful in differentiating between categories: message type (eg, a personal story, a news story, and a call to action) and the underlying perspective about suicide (ie, if the tweet applies a problem- and suffering- or solution- and coping-centered perspective). For each message type, we noticed that some tweets implicitly or explicitly frame suicide only as a problem or from an exclusively negative or suffering perspective (categories: suicidal ideation and attempts, suicide cases, and awareness), whereas other tweets implied that coping was possible or suggested ways of dealing with the problem (categories: coping stories and prevention).
4. Repeating step 2, we trained our best preliminary model to make new predictions for the 6 categories based on all labeled tweets. Each coder annotated a different set of tweets for each predicted label until we reached a minimum of 200 training examples for the smallest categories (suicidal and coping stories). This resulted in 2206 tweets in total.
5. To mitigate bias from the search terms we used to assemble our initial training set and to estimate the distribution of tweets across categories on Twitter, HM labeled a random sample of 996 tweets (initially 1000, with 4 were not labeled owing to a displaying error in the used spreadsheet). These were then added to the training set, resulting in a total sample of 3202 tweets.
6. After reviewing the entire training set, we finally refined the categories to allow for the following distinctions: for stories about coping and suicidal experiences, we differentiated the perspective from which an experience was described (first or third person), which experience was described (the one of a concerned or a bereaved individual), and whether a tweet was shared by news media or individual users. For reporting of cases, we distinguished tweets about individuals who had actually died by suicide from tweets about someone saving the life of an individual who was about to take his or her life. Finally, we organized the tweet categories according to 2 dimensions further described in the *Annotation Scheme* section.

### Annotation Scheme

#### Overview

The annotation scheme divides tweets into 12 categories, including 10 categories of interest and 2 irrelevant categories. Each category can be described in terms of two dimensions: the message type (eg, a personal story, a news story, and a call to action) and the underlying perspective about suicide (ie, if the tweet applies a problem- or solution-centered perspective). The perspective distinguishes messages that implicitly or explicitly frame suicide only as a problem or from an exclusively negative or suffering perspective from messages that imply that coping is possible or suggest ways of dealing with the problem. The organization of the tweet categories along these 2 dimensions is presented in Table 1. Detailed instructions for annotating tweets are provided in Multimedia Appendix 2. These include prioritization rules for how to deal with ambiguous tweets that may fit into more than one category.

**Table 1:** Annotation scheme of content categories organized along two dimensions: message type and underlying perspective about suicide.

Message type		Underlying perspective	
		Problem and suffering	Solution and coping
Personal experiences first or third person		Suicidal ideation and attempts^a^	Coping (Papageno)^a^
News about experiences and behavior		News suicidal ideation and attempts	News coping
Experience of bereaved		Bereaved negative	Bereaved coping
Case reports		Suicide cases (Werther)^a^	Lives saved
Calls for action		Awareness^a^	Prevention^a^
**Irrelevant^a^**			
	Suicide other	Murder-suicides, history, fiction, not being suicidal, and opinions	
	Off-topic^b^	Bombings, euthanasia, jokes, metaphors, and band or song names	

^a^The 6 main categories classified in machine learning task 1.

^b^Task 2 distinguished the off-topic category from all other categories (see *Classification Tasks*).

#### Description of Content Categories

For each message type (except for irrelevant messages), there is a category for more problem- or suffering-focused tweets and for more solution- or coping-focused tweets:

1. Personal stories describing the experience of an affected individual either in first- or third-person perspective:
  - Suicidal ideation and attempts: Personal stories about an individual’s negative experiences with suicidal thoughts, related suffering (eg, depression), suicidal communication and announcements, or suicide attempts without a sense of coping or hope
  - Coping: Personal stories about an individual’s experience with suicidal thoughts or a suicide attempt, with a sense of hope, recovery, coping, or mentioning an alternative to suicide. The sentiment does not have to be positive. A neutral tone or talking about difficult experiences with a sense of coping or mentioning recovery is sufficient. Previous research has suggested that such messages may have a Papageno effect.
2. News reports about suicidal experiences and behavior except cases, often about celebrities:
  - News suicidal ideation and attempts: About suicidal experiences without any mention of coping, including reports on suicidal ideation, suicide attempts, announcements of suicide, and someone being put on “suicide watch”
  - News coping: About attempted or successful coping with or recovering from a suicidal crises.
3. Tweets describing the experience of a person who has lost someone to suicide from the first- or third-person perspective:
  Bereaved negative: Describes the suffering or purely negative experience of a person who has lost someone to suicide, including depression, grief, and loss. These tweets necessarily refer to a suicide case but are labeled as bereaved as long as they focus on the experience of bereaved individuals.
  Bereaved coping: Describes the experience of a bereaved person with a sense of hope, recovery, or coping. The sentiment does not have to be positive. A neutral tone, or talking about difficult experiences with a sense of coping or mentioning recovery is sufficient.
4. Reports of a particular completed or prevented suicide cases, often news reports:
  Suicide cases: About an individual suicide or a timely or geographical suicide cluster. Suicide cases have priority over the definition criteria of other categories (except tweets focusing on bereaved individuals, which are always related to a suicide case). Previous research suggests that such messages on individual suicide deaths (especially about celebrities) may have a Werther effect.
  Lives saved: News report or personal message about someone saving a life. In contrast to prevention tweets, these lives are often being saved coincidentally.
5. Calls for action are general statements calling for actions addressing the problem of suicide and intending to spread problem awareness or prevention-related information:
  Awareness: Tweets intending to spread awareness for the problem of suicide, often focusing on high suicide rates or associations with bullying, racism, depression, and Veterans without hinting at any solution. These are often the reports of research findings or suicide statistics.
  Prevention: Tweets spreading information about a solution or an attempt to solve the problem of suicide, including prevention at an individual (eg, do not leave people alone in crisis situations) or public health level (eg, safety nets on bridges). Hinting at a solution or a way of dealing with the problem is sufficient. No specific action needs to be described. These tweets often include a helpline number. Announcements of prevention events and broad recommendations for actions also count—donations and prayers with a focus on a solution for suicide, being there for someone, telling people that they matter, taking a course about suicide prevention, and warning signs to watch out for.
6. Irrelevant, including messages that do not fit into any of the above categories:
  - Suicide other: Anything about actual suicide but not clearly related to any other above category, including murder-suicides, confident statements that something was not a suicide, convincing statements of not being suicidal, historical tweets about suicides that were a minimum of 40 years ago (eg, about the suicide of Hitler), movies, books, novels, and fiction about suicide.
  - Off-topic: Messages that use the term suicide in a context other than suicide. This includes messages on euthanasia, suicide bombing and suicide attacks, messages that are (suspected) jokes, irony, sarcasm, flippant remarks or really unclear in terms of authenticity, and messages that use suicidal or suicide to exaggerate an emotional experience (unclear if serious) or as a metaphor (eg, political, financial, or career suicide, suicide workout, and suicidal immigration policies), and messages about “suicidal animals” (eg, killed by car).

### Analysis

#### Software

Data analysis was performed using R for intercoder reliability, descriptive statistics, and figures (version 3.6.3, R Foundation for Statistical Computing). The main libraries used in R were tidyverse, caret, and DescTools [32-34]. For training deep learning models, we used Python 3.6 (Python Software Foundation). The main packages were the ktrain wrapper [35] for the deep learning library TensorFlow Keras [36] and the scikit-learn library [37] for term frequency-inverse document frequency (TF-IDF) and support vector machines (SVM). For links to code and data, refer to the *Data and Code Availability* section.

#### Text Preprocessing

We applied standard preprocessing strategies (eg, [38]) and replaced all URLs with a general marker token “*http*,” all mentions (tags of Twitter users) with “*@user*,” and lowercased all words. The latter allowed using the smaller, more resource-efficient Bidirectional Encoder Representations from Transformers (BERT)–lowercase model (see the *BERT-base* section). We kept emoji, stop words, and punctuation separated into single tokens, given that they can indicate the emotional connotation of a message (eg, expressing excitement or surprise [39] or frequent singular pronouns indicating suicidal ideation [15]). We report the effects of additional standard different preprocessing steps, namely removing digits, punctuation, stop words, and lemmatization, in Multimedia Appendix 1. The basic preprocessing strategy yielded the most consistently high-performance scores on the validation set and was therefore used for all analyses. After preprocessing, the mean length of tweets in our labeled data set was 25 tokens, the 95th and 99th percentile were 57 and 67 tokens, respectively (Figure S1 in Multimedia Appendix 1). On the basis of this, we used 80 tokens as the maximum sequence length for model input.

#### Classification Tasks

##### Task 1: 6 Main Categories

We trained our models to classify among categories with at least 200 tweets to allow sufficient training data. From the categories of interest, these were messages about (1) *personal experiences of coping*, (2) *personal experiences of suicidal ideation and attempts*, (3) *suicide cases*, (4) *awareness*, and (5) *prevention*. We assigned all tweets from smaller categories (suicidal and coping news, negative and coping experiences of the bereaved, and lives saved) to the category *suicide other*, which belongs to the larger category of *irrelevant* tweets. In this task, we did not differentiate between irrelevant tweets that were about suicide (suicide other) and off-topic tweets, which used the word suicide in some other way. Instead, we subsumed suicide other and off-topic tweets in the category (6) *irrelevant.*

##### Task 2: Detecting Content About Actual Suicide

This binary classification distinguishes tweets that are (1) *about actual suicide* in the meaning of someone taking their own life, from tweets that are (2) *off-topic*, that is, use the word suicide in some other context. In our annotation schema, this task therefore separates the off-topic category from all other categories. The resulting label predictions allow to estimate the total volume of tweets about actual suicide, thereby improving the total volume estimates only based on keyword searches.

#### Machine Learning Models and Model Training

##### Train-, Validation-, and Test Set

Before training models, we divided the data set of 3202 tweets into training (2049/3202, 63.99%), validation (512/3202, 15.99%), and test sets (641/3202, 20.02%), stratifying per tweet category to have a similar distribution in all sets. The training set was used for fitting the parameters of the classifier using 5-fold cross-validation. The validation set was used to tune the hyperparameters (eg, learning rate) and evaluate the model developed on the training data. After model training, we used the test set only once per model to estimate its ability to generalize to novel texts.

##### Majority Classifier

We used a naïve classifier that always predicts the majority class as a baseline to compare the other models.

##### TF-IDF and SVM

TF-IDF represents the text of tweets using weighted word *frequencies (f)*, which reflect how important a *term (t)* is to a *document (d, here a tweet)* in a corpus (all tweets). We slightly adjust the original formula for TF-IDF by adding 1 in the numerator and denominator, to ensure each word occurs at least once and prevent 0 division [40]: *tf-idf (t,d) = tf (t,d) × log([N +1]/[df + 1])*.

The resulting value increases proportionally to the number of times a word appears in the document and is offset by the number of documents in the corpus that contain the word. This helps to adjust the weight of uncommon words that are more important for distinguishing different documents from each other than words that occur in every single document. After building the TF-IDF representation, we trained a SVM classifier using all term values as features.

To identify the best TF-IDF representation and SVM classifier, we ran a grid search across the following dimensions. For TF-IDF, we (1) included only unigrams or unigrams+bigrams and (2) reduced the text to its n top features ordered by term frequency, where n ∈ {10,000; 25,000; 50,000}. For the SVM, we tested different hyper-parameters, namely (1) regularization parameter C ∈ {0, 1}, which determines the strength of the regularization and (2) class weight cw ∈ {balanced, none}, which determines whether the weights of the classes are automatically adjusted inversely proportional to class frequencies. We further tested (3) a linear and a radial basis function kernel and (4) decision function shapes one versus one and one versus rest. Optimal results were achieved including both unigrams and bigrams as text representation, 10,000 top features, and an SVM with C=0.82 in task 1 and C=0.46 in task 2, cw=balanced, a linear kernel, L2 penalty, and a one-versus-one decision function shape.

##### BERT Base

We used a transfer learning approach based on a pretrained BERT-base-uncased model [41]. BERT is an autoencoding deep contextual language representation model developed by Google AI, which has 12 transformer layers, 12 self-attention heads, and a hidden size of 768. It is designed to pretrain bidirectional representations of word sequences, that is, it learns from both the left-side and right-side context of a word in all of its layers. BERT was pretrained with masked language modeling: a percentage (approximately 15%) of words in the sentence is randomly masked, and the model tries to predict the masked words from the sequence of other words. BERT was further trained to predict the next sentence from the previous sentence in the data.

##### XLNet Base

A known limitation of BERT is that it neglects the dependence between the different masked words in a sentence. When predicting a word from a sequence that does not include the other masked words, BERT lacks information about the dependence between the masked words and the predicted word. Unlike autoregressive models, BERT further predicts all masked words simultaneously, and thus lacks some information about the order of words. XLNet [42] has a similar architecture as that of BERT but addresses these shortcomings through permutation language modeling, predicting each word from all possible permutations of other words in the sentence. It thereby improves both on previous autoregressive models by using all words in the sentence and on BERT by considering the order dependence of words. In addition, it incorporates some techniques from Transformers-XL [43], which also allows it to learn from the longer context before each word (relative positional encoding and segment recurrence mechanism).

##### Fine-tuning of BERT and XLNet

We fine-tuned the pretrained BERT-base uncased model and the XLNet-base model to our training data set as in the study by Liu et al [16], added one dense output layer to reduce the dimensions of the model’s last layer to the number of labels in the classification task, and trained all the parameters simultaneously. We ran a hyper-parameter search to determine good learning rate (LR) candidates and subsequently tested each LR by running 3 experiments with different seeds ∈ {1,2,3}. We aimed to find the maximal LR associated with a still-falling loss (before the loss diverging) by training for 5 epochs with learning rates ∈ {2e-5, 3e-5, 5e-5}. The reported results for BERT in Task 1 (6 classes) were the result of fine-tuning with a LR=2e-5 for 7 epochs and seed=1. The results for task 2 (about actual suicide) were based on a BERT model trained with a LR=1e-5, 10 epochs, and seed=1. The reported results for XLNet were based on model training with LR=2e-5, 8 epochs, and seed=1 in both tasks.

### Metrics for Comparing Machine Learning Models

We used various evaluation metrics to compare different machine learning models. *Accuracy* indicates the percentage of correct predictions (true positive and true negative). It is a global metric calculated for all the classes in a data set. In data sets with large class imbalances, it can be high even if it always predicts only the majority class (eg, the irrelevant category in task 1). In this case, the model may not have learned anything despite its high accuracy. *Precision* indicates the proportion of correct “positive” predictions out of all predictions; for example, how many of all predicted coping tweets were actually labeled as coping tweets by human raters. *Recall* indicates the proportion of all “true” cases (eg, all actual coping tweets) that the model detects. The *F*_1_-score is the harmonic mean between precision and recall (*F*_1_ = 2 × [precision × recall]/[precision + recall]). Precision, recall, and *F*_1_-scores were calculated for each category and can be averaged across classes to produce a macroaverage. For category-specific precision and recall, we provide 95% binomial CIs calculated using the Clopper-Pearson method.

To compare models, we report macroaverages of model performance scores for both the validation and test sets, that is, we calculate the mean of the performance measures of each class to have an aggregate measurement robust to class imbalance. Although good scores on the training set indicate that the model has learned patterns existing in the training set, good scores on the test set additionally indicate an ability to generalize to novel data.

For determining the model to make predictions for a follow-up study (Niederkrotenthaler et al, unpublished data, May 2022; see Multimedia Appendix 1 for details), we decided a priori that we would prioritize precision over recall for task 1, which aims to identify specific categories of tweets. The rationale behind this is that our follow-up study focused on identifying specific Twitter signals (ie, the percentage of coping tweets) that are associated with suicide cases and helpline calls. In such a situation, a false negative is less costly than a false positive, that is, missing a tweet is less costly than falsely including a tweet in a certain category. Prioritizing precision ensures that we only count a tweet when it belongs to a category with a high probability. Furthermore, because of the large number of tweets, the proportion assigned to each category should accurately reflect the true proportion, even if not all the tweets are recognized. In contrast, task 2 makes predictions that aim to capture the entire discussion about actual suicides on Twitter. When choosing the best model for task 2, we focused on the *F*_1_-score. Here, we aimed to capture the total volume of tweets about suicide as fully as possible, as well as at accurate predictions at the tweet level. False positives are less critical as a problem in task 2 than in task 1, because we look at total tweet volume and do not try to distinguish between the specific effect of a certain tweet category. This is best captured with the *F*_1_-score, which balances recall and precision. In any case, none of these a priori decisions had consequences for our results, given that BERT and XLNet performed very similarly and much better than the other models.

### Evaluating Reliability and Face Validity of Model Predictions for BERT

#### Comparing Model and Human Interrater Reliability

To compare the models’ reliability to human interrater reliability on novel data, we made predictions using one of the best models (BERT) for tweets from the full data set that were not part of the labeled data set. We selected 150 tweets per predicted label for each of the 5 relevant main categories. In all, 2 independent human coders manually labeled these tweets until we reached at least 80 tweets per main category. The final set of 750 labeled tweets comprised the reliability data set.

#### Face Validity Check With the Predictions Data Set

For a face validity check and a follow-up study [30], we estimated the daily volume of tweets per category that Twitter users may have been exposed to between January 1, 2016, and December 31, 2018. For this, we created a data set with the same keywords and exclusion terms as the machine learning data set but including retweets (to account for the full volume) and for a shorter period of 3 years (determined by the follow-up study [30]). This resulted in 7,150,610 tweets in English from users in the United States. We used the machine learning model BERT to predict the category labels for these tweets and calculated the daily percentage of tweets per category. We refer to this data set with model predictions for approximately 7 million tweets as the prediction data set. As a face validity check, we plotted the time series of tweet volumes per category and identified the events associated with the largest frequency peaks. We investigated word frequencies on these days, read the tweets containing the most frequent terms, and Googled these terms plus the date, or the tweet in quotes, to find (news) reports about the event. The follow-up study [30] investigated the associations of these daily tweet volumes with suicide cases and helpline calls in the United States. It has access to suicide case data from the Centers for Disease Control and Prevention and call data from the United States suicide prevention lifeline for the years 2016 to 2018, which was the reason for estimating tweet volumes for this period.

**Figure 2:**
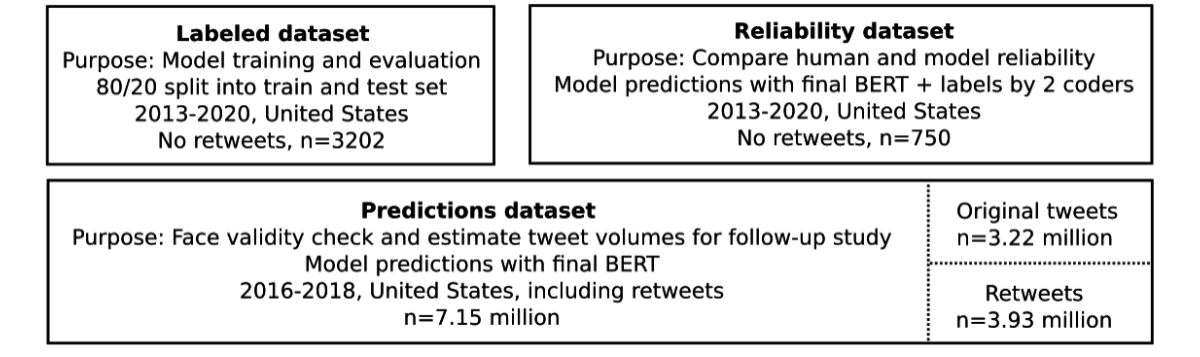
Overview of characteristics of data sets. Each box describes the purpose of the data set, further details on how it was used or created, and the sample size. Only the predictions data set includes retweets, as it aims to capture the full volume of tweets posted on a given day. BERT: Bidirectional Encoder Representations from Transformers.

Properties of all data sets used in this study, including the labeled machine learning data set and those for comparing model and human performance and the face validity check are depicted in Figure 2.

## Results

### Frequency of Tweets per Category

Table 2 displays the proportion of tweets per main category in our labeled data set and in 2 different samples used to estimate the natural frequency of categories on Twitter. First, we used a subsample of the labeled data set of 1000 randomly selected tweets (ie, selected without keywords or model predictions, 996 after 4 labeling; see *Creating the Annotation Scheme and Labeled Data Set* section) to estimate the frequency of original tweets, without counting retweets. For the second estimate, we used predictions by the best model (BERT) to label tweets in the prediction data set, which included retweets. The 2 estimates were similar for suicidal ideation and attempts and suicide cases.

The percentages per category in Table 2 demonstrate that we managed to include proportionally more rare tweet categories, such as coping and suicidal ideation stories in our training set. Nevertheless, irrelevant tweets, particularly off-topic tweets, still make up a majority of tweets in our data set.

**Table 2:** Distribution of tweets across categories for manual labels and model predictions.

Category label			Total labeled sample (n=3202)		Subset of labeled tweets, randomly selected (n=1000)		Estimated frequency in predictions data set (including retweets; n=7.15 million), n (%)^a^		
							Task 1		Task 2
Suicidal ideation and attempts, n (%)			284 (8.87)		63 (6.33)		367,135.56 (5.13)		5,471,499 (76.52)
Coping, n (%)			205 (6.4)		26 (2.71)		90,328.99 (1.26)		5,471,499 (76.52)
Awareness, n (%)			314 (9.81)		126 (12.54)		1,577,650 (22.06)		5,471,499 (76.52)
Prevention, n (%)			457 (14.27)		71 (7.13)		1,109,223.6 (15.51)		5,471,499 (76.52)
Suicide cases, n (%)			514 (16.05)		129 (12.95)		1,155,277.92 (16.16)		5,471,499, (76.52)
Irrelevant, n (%)			1428 (44.5)		581 (58.33)		2,850,994 (39.88)		5,471,499 (76.52)
**Subcategories of irrelevant, n (%)**									
	News suicidal	68 (2.12)		20 (2.01)		2,850,994 (39.88)		5,471,499 (76.52)	
	News coping	27 (0.84)		5 (0.5)		2,850,994 (39.88)		5,471,499 (76.52)	
	Bereaved negative	34 (1.06)		7 (0.7)		2,850,994 (39.88)		5,471,499 (76.52)	
	Bereaved coping	34 (1.06)		5 (0.5)		2,850,994 (39.88)		5471499 (76.52)	
	Live saved	13 (0.41)		2 (0.2)		2,850,994 (39.88)		5,471,499 (76.52)	
	Suicide other	440 (13.74)		206 (20.68)		2,850,994 (39.88)		5,471,499 (76.52)	
	Off-topic	812 (25.36)		336 (33.73)		2,850,994 (39.88)		1,679,111 (23.48)	

^a^For the predictions data set: Absolute values and percentages were weighted (ie, divided) by the model’s recall (proportion of all true cases the model detects). Sample values (n) and percentage for the irrelevant category were calculated by subtracting the sum of all other categories from the total sample size and 100, respectively. If several cells contain the same values, this is because they were subsumed to one higher-level category (*irrelevant* in task 1, *about suicide* in task 2) in the respective classification task.

### Model Performance

#### Task 1: 6 Main Categories

Performance scores averaged across all 6 tweet categories (Table 3) show that all deep learning models performed very similarly and substantially better than the TF-IDF and SVM approaches. However, TF-IDF and SVM were clearly better than a naïve majority classifier. It reached scores from 0.61 to 0.66, which were nearly identical on the validation and test sets. For BERT and XLNet, all scores were at or above 0.70, and only 0.1 to 0.3 lower on the test than the validation set, indicating a good ability to generalize to new tweets. The macroaverage performance scores in all 5 runs for BERT and XLNet were approximately 10% higher than the TF-IDF and SVM macroaverages (Table S2 in Multimedia Appendix 1).

**Figure 3:**
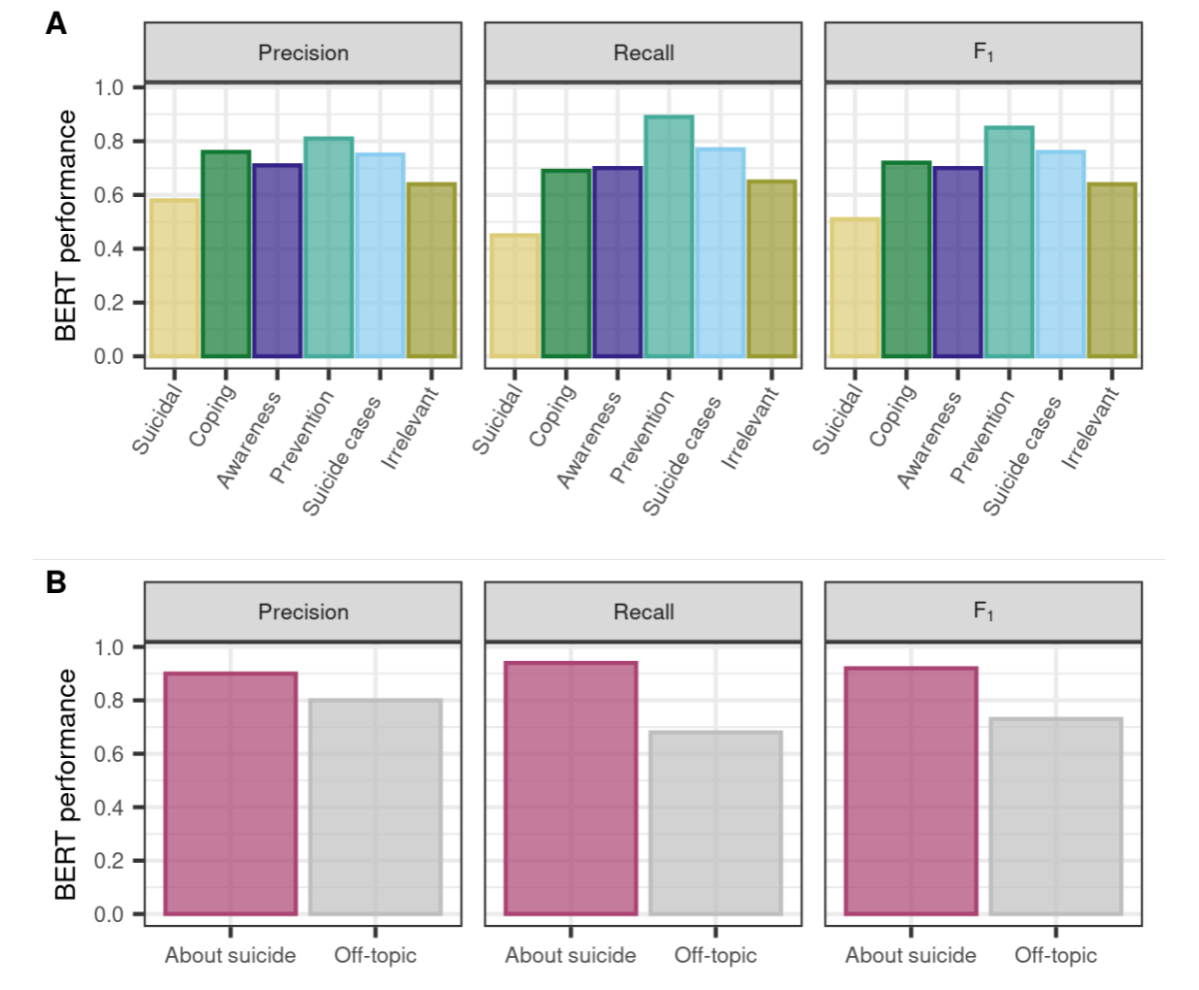
Performance scores per category for Bidirectional Encoder Representations from Transformers (BERT) for the 6 main categories (A) and for tweets about actual suicide versus off-topic tweets (B).

Given that the macroaverage performances were substantially lower for the majority classifier, we focused on the 3 other models for intraclass scores (Table 4 and Figure 3). To choose a model for making predictions, we focused on *F*_1_-scores and precision (see the section on evaluation metrics). *F*_1_-scores were higher for BERT and XLNet than for TF-IDF and SVM for all relevant categories, with clear differences for some categories (suicidal, coping, and awareness) and very small differences for others (suicide cases and prevention). For BERT and XLNet, *F*_1_-scores were almost identical for all categories. BERT yielded higher precision for coping and prevention tweets, 2 crucial categories for a follow-up publication (Niederkrotenthaler et al, unpublished data, May 2022; see Multimedia Appendix 1 for details). Therefore, we chose BERT as the model to make predictions for further analyses. It should be noted here that CIs are quite large because of the limited size of the test set per class and entirely overlap for BERT and XLNet and somewhat overlap for most categories with TF-IDF and SVM. Nonetheless, the performance scores in the 5 runs of BERT and XLNet were higher than those of TF-IDF and SVM in almost all cases for all relevant categories (Table S3 in Multimedia Appendix 1). Only in the case of precision for prevention tweets, TF-IDF and SVM performed similarly well in 3 out of 5 runs.

Overall, BERT correctly classified 73% of the tweets in the test set. *F*_1_-scores lay between 0.70 and 0.85 for the different categories of interest (Table 4 and Figure 3), with the exception of the suicidal ideation and attempt category, with an *F*_1_-score of 0.51. More specifically, recall for suicidal ideation and attempt was relatively low (0.45), indicating difficulties in detecting all such tweets, whereas precision was higher, with 0.58. All performance scores were particularly good (>0.81) for prevention tweets and high for tweets about suicide cases (>0.75). For coping tweets, BERT achieved very high precision (0.76) but lower recall (0.69), which resembles the pattern observed for suicidal tweets. Performance scores for awareness tweets were approximately 70%.

**Table 3:** Macroaveraged performance metrics and accuracy cross all 6 categories on the validation and test set.

Model	Validation set (n=513)					Test set (n=641)			
	Precision	Recall	*F*_1_	Accuracy	Precision		Recall	*F*_1_	Accuracy
Majority classifier	0.07	0.17	0.10	0.45	0.07		0.17	0.10	0.44
TF-IDF^a^ and SVM^b^	0.61	0.63	0.62	0.66	0.61		0.65	0.62	0.66
BERT^c,d^	0.73	0.71	0.71	0.76	0.72		0.69	0.70	0.73
XLNet^d^	0.74	0.73	0.73	0.77	0.71		0.71	0.71	0.74

^a^TF-IDF: term frequency-inverse document frequency.

^b^SVM: support vector machine.

^c^BERT: Bidirectional Encoder Representations from Transformers.

^d^Given that the performance of both deep learning models with fixed seeds and parameters varied slightly from run to run owing to internal segmentation, we ran these models 5 times. We report the average of all 5 runs in this section and include the metrics for each individual run in Table S2, in Multimedia Appendix 1.

**Table 4:** Intraclass performance metrics on the test set.

Category	TF-IDF^a^ and SVM^b^				BERT^c,d^				XLNet^d^		
	Precision (95% CI)	Recall (95% CI)	*F*_1_	Precision (95% CI)		Recall (95% CI)	*F*_1_	Precision (95% CI)		Recall (95% CI)	*F*_1_
Suicidal ideation (n=57)	0.32 (21.93-43.58)	0.44 (30.74-57.64)	0.37	0.58 (43.25-73.66)		0.45 (32.36-59.34)	0.51	0.60 (46.11-74.16)		0.54 (40.66-67.64)	0.55
Coping (n=42)	0.44 (31.55-57.55)	0.64 (48.03-78.45)	0.52	0.76 (59.76-88.56)		0.69 (52.91-82.38)	0.72	0.71 (54.80-83.24)		0.74 (57.96-86.14)	0.73
Awareness (n=63)	0.65 (51.60-76.87)	0.62 (48.80-73.85)	0.63	0.71 (58.05-81.80)		0.70 (56.98-80.77)	0.70	0.69 (56.74-79.76)		0.74 (62.06-84.73)	0.72
Prevention (n=91)	0.83 (74.00-90.36)	0.82 (73.02-89.60)	0.83	0.81 (71.93-88.16)		0.89 (80.72-94.60)	0.85	0.82 (72.27-88.62)		0.87 (78.10-93.00)	0.84
Suicide cases (n=103)	0.70 (60.82-78.77)	0.74 (64.20-81.96)	0.72	0.75 (65.14-82.49)		0.77 (67.34-84.46)	0.76	0.78 (68.31-85.52)		0.75 (65.24-82.80)	0.76
Irrelevant (n=285)	0.74 (67.78-79.18)	0.63 (57.27-68.77)	0.68	0.64 (57.76-69.11)		0.65 (59.06-70.45)	0.64	0.68 (61.96-73.46)		0.64 (57.99-69.44)	0.66

^a^TF-IDF: term frequency-inverse document frequency.

^b^SVM: support vector machine.

^c^BERT: Bidirectional Encoder Representations from Transformers.

^d^Scores are averages across 5 model runs for BERT and XLNet. Table S3 in Multimedia Appendix 1 shows separate runs.

#### Task 2: About Actual Suicide

Best performances for separating tweets about actual suicide from off-topic tweets (Table 5) were observed with BERT. However, XLNet performances were very similar, with largely overlapping CIs. With TF-IDF and SVM, recall for about suicide tweets and precision for off-topic tweets were significantly lower than the deep learning scores, whereas precision for about suicide and recall for off-topic was not significantly different. The model with overall highest scores, BERT, correctly labeled 88.5% of tweets as about suicide versus off-topic, with very similar scores on the validation and test sets. *F*_1_-scores for about suicide versus off-topic tweets in the test set were 0.92 and 0.73, respectively (Table 6). All metrics were at least 10% higher for tweets about suicide than for the off-topic tweets. In particular, recall was very high for tweets about suicide (94%), which indicates that volume estimates for tweets related to suicide would be quite complete. The precision for tweets about suicide was 90%, indicating that positive predictions of the model were very reliable.

**Table 5:** Macroaveraged performance metrics and accuracy for task 2 (about suicide vs off-topic) on the validation and test sets.

Model	Validation set (n=513)					Test set (n=641)			
	Precision	Recall	*F*_1_	Accuracy	Precision		Recall	*F*_1_	Accuracy
Majority classifier	0.37	0.50	0.43	0.75	0.37		0.50	0.43	0.75
TF-IDF^a^ and SVM^b^	0.74	0.77	0.75	0.80	0.75		0.77	0.76	0.81
BERT^c^	0.85	0.81	0.83	0.88	0.85		0.81	0.83	0.88
XLNet	0.84	0.78	0.81	0.87	0.83		0.80	0.81	0.87

^a^TF-IDF: term frequency-inverse document frequency.

^b^SVM: support vector machine.

^c^BERT: Bidirectional Encoder Representations from Transformers.

**Table 6:** Intraclass performance metrics for deep learning models in task 2 (about suicide vs off-topic) on the test set.

Test set and model	About suicide (n=478)				Off-topic (n=163)		
	Precision (95% CI)	Recall (95% CI)	*F*_1_	Precision (95% CI)		Recall (95% CI)	*F*_1_
TF-IDF^a^ and SVM^b^	0.89 (85.74-91.71)	0.85 (80.96-87.64)	0.87	0.60 (53.03-67.49)		0.69 (61.63-76.30)	0.65
BERT^c,d^	0.90 (87.42-92.81)	0.94 (91.64-96.07)	0.92	0.80 (71.62-85.67)		0.68 (60.35-75.17)	0.73
XLNet^d^	0.90 (87.12-92.59)	0.93 (90.68-95.38)	0.92	0.76 (68.60-83.06)		0.67 (59.72-74.60)	0.71

^a^TF-IDF: term frequency-inverse document frequency.

^b^SVM: support vector machine.

^c^BERT: Bidirectional Encoder Representations from Transformers.

^d^Scores are averages across 5 model runs for BERT and XLNet. Table S5 in Multimedia Appendix 1 shows separate runs.

### Comparing Model and Human Interrater Reliability

#### Task 1: 6 Main Categories

The interrater reliability (Cohen κ) for the 6 main categories was 0.70 (95% CI 0.67-0.74) between 2 human coders and 0.60 (95% CI 0.56-0.64) and 0.63 (95% CI 0.59-0.67) between each human coder and the BERT model, respectively. The lower agreement with BERT compared with between humans was mainly driven by the irrelevant class. Excluding it from analysis yielded κ=0.85 (95% CI 0.82-0.89) between human raters and κ=0.81 (95% CI 0.77-0.85) and 0.80 (95% CI 0.76-0.84) between BERT and each human rater. These overlapping CIs indicate a nonsignificant difference and show that BERT achieved near human-level accuracy for the relevant categories.

**Figure 4:**
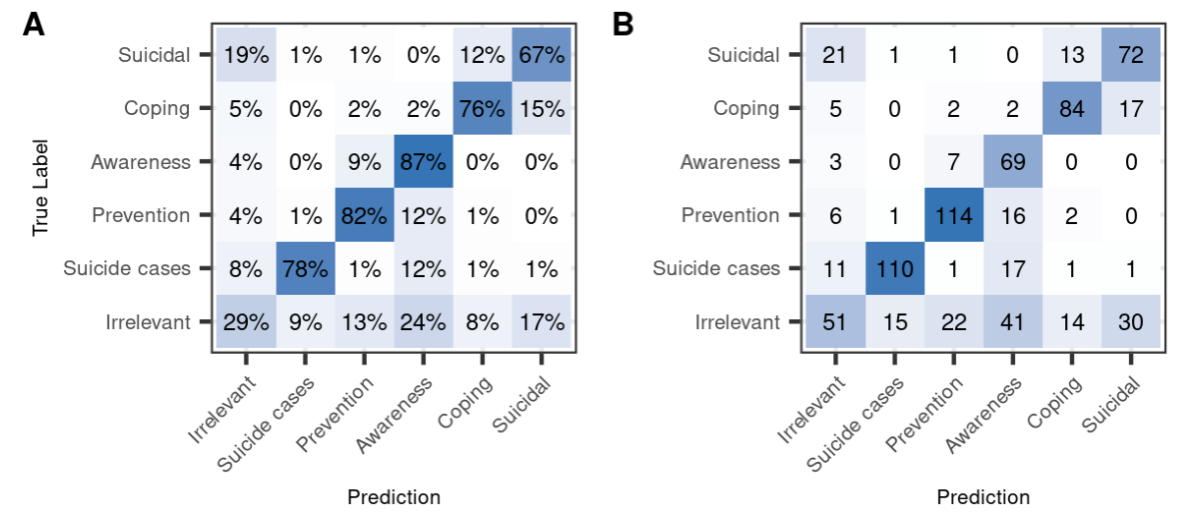
Confusion matrix of true and predicted labels in the reliability data set. (A) percentages and (B) count of tweets per true and predicted category. The diagonal from bottom left to top right represents correct predictions. True labels are labels by coder 1, and predicted labels are by Bidirectional Encoder Representations from Transformers (BERT).

Precision and recall comparisons between model and human performance per tweet category are shown in Table S6 in Multimedia Appendix 1, and the confusion matrix for coder 1 and BERT is shown in Figure 4. First, we report the metrics for the model versus each coder, with the coder as the ground truth. Second, we report the same metrics for coder 2 compared with coder 1 as the ground truth. Model precision was clearly lower than between-human precision for *suicidal ideation and attempts* and *awareness* messages, more comparable for *coping stories*, and very similar for *prevention* and *suicide case tweets.* Recall is clearly higher between human raters for *suicidal and coping* stories and similar for *suicide cases*. For awareness and prevention tweets, the model actually achieves better recall than human coders. Thus, the model seems quite good at detecting awareness tweets but is not very precise in return.

#### Task 2: About Actual Suicide

When categorizing tweets as being about actual suicide versus off-topic, human interrater reliability was κ=0.44 (95% CI 0.29-0.58) compared with κ=0.15 (95% CI −0.07 to 0.37) and κ=0.21 (95% CI −0.01 to 0.44) between each coder and BERT. These low κ coefficients were mainly driven by low performances for the irrelevant off-topic category between both human coders (coder 1-coder 2: precision=0.52, recall=0.44, and *F*_1_=0.48), which were even lower when comparing human to model labels (coder 1-BERT: precision=0.26, recall=0.13, and *F*_1_=0.17; coder 2-BERT: precision=0.39, recall=0.16, and *F*_1_=0.23). In contrast, the performance for the suicide category was very high when comparing human labels (precision=0.96, recall=0.97, and *F*_1_=0.96), as well as when comparing human and model labels (coder 1-BERT: precision=0.94, recall=0.98, and *F*_1_=0.96; coder 2-BERT: precision=0.94, recall=0.98, and *F*_1_=0.96). This shows that the 2 coders and the model agreed which tweets were about actual suicide and detected most tweets that the other coder had labeled as about suicide. However, they agreed less when judging whether a tweet was not about actual suicide, hinting at the inherent difficulty of judging whether something is serious, sarcastic, or metaphorical. In any case, for future studies correlating tweets about suicide with behavior in the population, only the about suicide category, which can be reliably detected by humans and the machine learning model, is relevant.

#### Error Analysis

Figure 4 shows the confusion matrix of the true and predicted labels for BERT for the 6 main categories in the reliability data set. Most misclassifications were predictions of the label irrelevant. Such false negatives are less problematic than misclassifications between relevant categories, as we prioritized precision over recall. Among the relevant categories, there were 5 cases in which coder 1 and the model labeled >9 but a maximum of 15% of tweets differently: (1 and 2) confusions between coping and suicidal tweets in both directions, (3 and 4) confusions between awareness and prevention tweets in both directions, and (5) tweets about suicide cases misclassified as awareness tweets.

For the 13 “true” suicidal labels where coder 1 and the model disagreed (12% of the 108 suicidal tweets), only 2 of the model labels were clear errors, all other tweets were ambiguous. Coder 2 and the model agreed on the coping label for one-third of these tweets (4/13, 31%), indicating the difficulty of clearly separating personal stories about suicidal ideation and coping even for humans. The model’s label more closely matched the category definition than coder 1’s label in at least 5 of the 13 cases (38.4%). Many of the ambiguous tweets described suicidal ideation in the past, implicitly hinting that the suicidal phase was over when the tweet was written. Out of 17 misclassifications of coder 1’s coping tweets (15% of the 110 coping tweets), coder 2 and the model agreed on the suicidal label in 6 cases (35.2%), suggesting that many of these misclassified tweets were ambiguous. Although 12 of these 17 (71%) misclassifications were actual model errors, most of them were understandable, given that coping was described implicitly by means of suicidal ideation in the past or that strong suicidal ideation was expressed along with a way in which the person deals with it.

Misclassifications of awareness as prevention tweets (7/79, 9%) were errors by coder 1 rather than the model in 4 of 7 cases (57%), indicating that model performances are higher for awareness tweets as the scores in Table 3 suggest. In contrast, when the model labeled prevention tweets as awareness, these were mostly clear mistakes (only 3 out of 16, 19%, were ambiguous errors by the coder). Finally, suicide cases mislabeled as awareness were mostly actual errors by the model, but ambiguous in 4 out of 17 cases (24%) and actually correct in 2 out of 17 cases (12%).

There was further a strong confusion between stories of suicidal ideation and a particular type of irrelevant tweet: tweets manually labeled as not serious or unclear if serious (dimension 3 in Multimedia Appendix 2). Of all tweets predicted to be personal stories of suicidal ideation or attempts, 10.8% (13/120) were not serious or were unclear, compared with only 1%-4% in the other predicted categories. Of all nonserious or unclear tweets, 45% (18/40) were correctly classified as irrelevant, 32% (13/40) were wrongly classified as suicidal compared with 3%-10% wrongly assigned to the other categories. The 13 (32%) nonserious tweets that were misclassified as suicidal included 4 exaggerations, 2 sarcastic remarks, 2 tweets with song lyrics or band names with the terms suicidal ideation or thoughts, 1 metaphoric use, and 2 statements about not being suicidal.

#### Face Validity Check With Daily Time Series Peaks per Category

**Figure 5:**
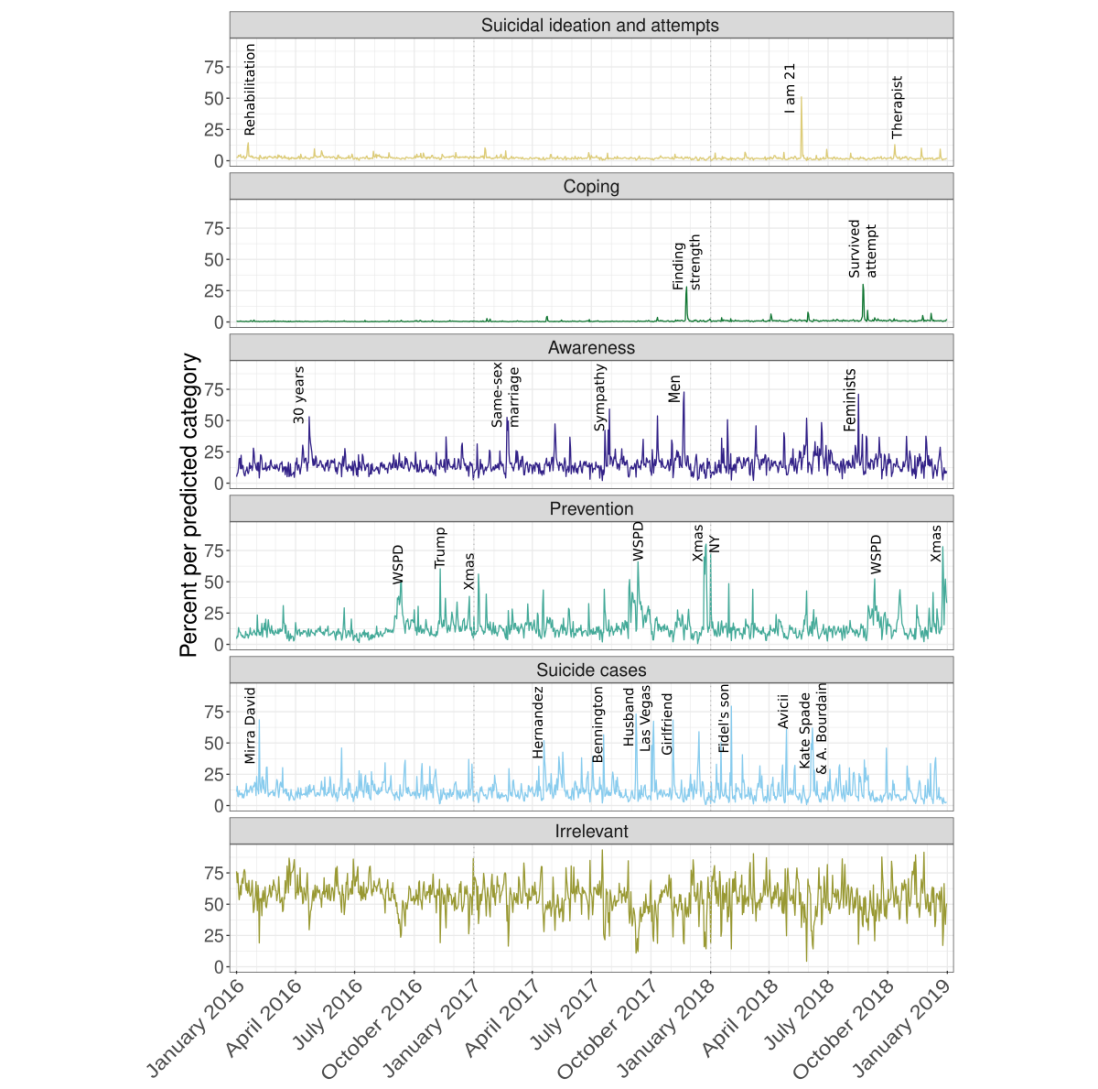
Daily percent of tweets per predicted category in the predictions data set (n=7.15 million). The daily value subsumes original and retweets per category. Key words for event peaks are explained in the main text.

Figure 5 illustrates the daily percentage of tweets in each predicted category in the prediction data set. As a face validity check, we identified the events that were mainly associated with each of the largest peaks in the time series of tweets per predicted category. These events are labeled with the following keywords in Figure 5: (1) S*uicidal ideation and attempts*: rehabilitation—4000 retweets of a rather sarcastic tweet from someone getting “punished” in rehabilitation because he said “fucking lit” that another patient was about to commit suicide; I am 21—a personal story retweeted 2200 times, describing someone’s successful journey from a difficult childhood, through a long suicidal crisis, to a University degree and a full time job, falsely labeled as suicidal, as it describes not just a suicidal crisis, but also coping; therapist—900 retweets of someone reporting no suicidal thoughts as their therapist asked, although they have them very frequently. (2) C*oping*: finding strength—approximately 6000 retweets of a story of someone finding strength 3 weeks after a suicide attempt; survived attempt—a marine corps Veteran with posttraumatic stress disorder tweets about his survived suicide attempt, approximately 5000 retweets. (3) *Awareness*: sympathy—a tweet saying people who died by suicide need care while still alive, rather than sympathy when they are dead, retweeted 3500 times; men—7000 retweets of a tweet mentioning that suicide is the largest cause of young men; feminists—6000 retweets of a tweet saying that feminists who want equality should also consider that boys are double as likely to die from suicide than girls; 30 years—retweets and discussion of a federal data analysis results that suicide in the United States had risen to the highest levels in nearly 30 years; same-sex marriage—many tweets on research finding that suicide rates drop after legalization of same-sex marriage. (4) *Prevention*: Trump—increased calls to suicide hotline after Trump’s election. (5) *Suicide cases* [44]: Aaron Hernandez (American football player); Chester Bennington (singer of Linkin Park); husband—retweets of tweet by a woman remembering her husband’s suicide; Las Vegas—many retweets of a reply correcting a tweet by Trump, by stating that the shooter killed himself; girlfriend—many retweets of a tweet about a girlfriend who killed herself; Fidel’s son—Fidel Castro Diaz-Balart; NY—new year; WSPD—World Suicide Prevention Day; and Xmas—Christmas.

For coping, prevention, and suicide case tweets, all highly frequent tweets were correctly classified tweets. Both highly shared coping tweets were from individuals who had survived a suicide attempt. Prevention peaks were related to the yearly World Suicide Prevention Day, to increased prevention efforts around Christmas and the New Year, and to increased lifeline calls after Trump’s election [45]. All the identified peaks of tweets about suicide cases were related to actual instances of someone taking their own life. For awareness tweets, all but one peak were driven by actual awareness tweets. This single tweet (labeled same-sex marriage in Figure 3) was ambiguous, as it reported a research finding similar to a typical awareness tweet, but the finding was somewhat prevention related. Most awareness peaks were driven by tweets that cite a statistic about suicides. Of the tweets driving the 3 largest peaks in the suicidal category, only 1 was clearly suicidal ideation, another was a somewhat cynical tweet about someone else wanting to commit suicide, and a third was a clear confusion with an actual coping tweet. Thus, this face validity check reflects the high precision of the model for prevention, awareness, and coping tweets, as well as the lower performance for suicidal ideation and attempt tweets.

## Discussion

### Overview of This Study

Owing to the effort required for manual annotation of texts, previous research on media and suicide prevention was limited by small sample sizes or by data sets put together using keyword search. Keywords either capture only a particular type of text (eg, containing celebrity names) or lump together a variety of different texts that contain broad search terms (eg, “suicide” [2,10-12]) In addition, research on the correlation of social media content with suicide cases in the population remains extremely scarce [9-13]. This study extends media research on suicide prevention by focusing on a broad range of suicide-related content on social media and by developing a reliable and efficient content labeling method based on machine learning, enabling fine-grained analysis of large data sets. We first developed a comprehensive annotation scheme for suicide-related content that includes new content types more typical on social than traditional media, such as personal stories of coping or suicidal ideation, or calls for action addressed at follower networks. On the basis of this systematic labeling scheme, we then tested the ability of different machine learning algorithms to distinguish 5 content types that seem particularly relevant based on previous research [2,4,6,30]. We further applied these methods to separate tweets about actual suicide, that is, in the meaning of someone taking their own life, from tweets that use the word suicide in some other way or context (binary classification). Our results for these 2 classification tasks show that machine learning methods, particularly deep learning models, achieve performances comparable with both human performance and with state-of-the-art methods in similar tasks [24,46].

This study is one of the first to automatically classify social media data other than suicidal ideation into categories relevant for suicide prevention. Only 3 studies, 2 (67%) of which used the same data set, have previously applied machine learning to distinguish specific types of social media posts other than suicidal ideation [9,24,25]. We extend these studies in several ways. Rather than classifying emotions in tweets about specific celebrities [9] or using a relatively small set of 816 tweets put together with a focus on suicidal ideation and celebrity names [24,25], we trained models to categorize any type of tweets containing suicide-related terms in a much larger data set than in previous studies. Furthermore, our larger data set enabled us to use deep learning models that can account for differences in the meaning of words across contexts, rather than only considering word frequencies. Finally, our annotation scheme introduced more fine-grained and particularly prevention-relevant categories. Specifically, it includes personal coping stories, for which some research on traditional media suggests preventive effects [4-6], and distinguishes awareness from prevention-focused tweets [27].

### Principal Findings

Regarding the machine learning results, pretrained deep learning models fine-tuned to our data clearly outperformed a naïve majority classifier and a linear SVM classifier based on the word frequency representation TF-IDF. BERT and XLNet achieved *F*_1_-scores of 0.70 and 0.71 in the 6-category classification and 0.83 and 0.81 in the binary about suicide versus off-topic classification in the test set. These scores were only slightly lower or even identical to those in the validation set, indicating good generalization to novel data. The clear advantage of deep learning models over TF-IDF and SVM suggests that there is crucial information about meaning in the context of words beyond what mere word frequencies can capture in tweets about suicide. Performance of the deep learning models was better than the more traditional approaches, but was very similar between BERT and XLNet. Advantages of XLNet over BERT include its ability to learn from long contexts and to consider dependencies between all words in the sentence. It seems that these advantages cannot be fully exploited given the limited number of words in tweets.

The 6 investigated tweet categories separated five important categories, including personal stories about either (1) coping or (2) suicidal ideation and attempts, calls for action that spread (3) problem awareness or (4) prevention-related information and (5) tweets about suicide cases, from other tweets (6) irrelevant to this categorization. The performance scores per category were nearly indistinguishable for BERT and XLNet. The model that performed better depended on the metric and the category and varied between model runs. BERT had slightly higher precision than XLNet for 2 important categories for a follow-up publication Niederkrotenthaler et al, unpublished data, May 2022; see Multimedia Appendix 1 for details) and was therefore chosen as the model to make predictions and test reliability. Although our data set included a much broader set of tweets than previous studies focusing on similar prevention-related tweet categories [24,25], our machine learning performances were comparable or better than in these previous studies, with the exception of the suicidal ideation category.

In general, BERT and XLNet were better at classifying tweets that are also easier to distinguish for humans, including more homogeneous classes such as prevention and suicide cases. These often included similar keywords, such as prevention, hotline, lifeline, or the phrase “committed/commits suicide” (see word clouds in Multimedia Appendix 1). For these categories, BERT performance was very similar to the human interrater performances. BERT and human performance were also comparable for coping stories. The model’s performance was lower only for more subjective classes such as suicidal ideation and attempt stories. Error analysis suggests confusions with sarcastic, joking, exaggerated, or metaphorical uses of the word suicide as one part of the explanation. Such nonserious messages are difficult to distinguish from genuine suicidal ideation for both the model and humans. The gap between human and model performance is the largest for suicidal tweets, suggesting that this distinction is even more difficult for the model. Both humans and the model missed many ambiguous expressions of suicidal ideation (low recall). In contrast, between-human precision is much higher than the model’s precision. This shows that there are many suicidal ideation tweets that humans can clearly identify, whereas model reliability can still be improved for these types of tweets.

The analysis of the most common model errors demonstrates that the mistakes were mostly not trivial. Most confusions of suicidal and coping tweets by the model were tweets in which the feelings of the tweet author were quite ambiguous. This suggests that much higher performance scores are difficult to achieve for these personal stories about suicide. Nonetheless, for tweets about suicidal ideation in the past, which implicitly express that coping occurred, there may be room for improvement through adding more training examples. Furthermore, the error analysis suggests possible improvements for tweets regarding prevention and suicide cases. In contrast, the model actually helped detect errors by the human coder for awareness tweets labeled as prevention.

When distinguishing tweets about actual suicide from off-topic tweets, the model achieved excellent performance scores, particularly for tweets about actual suicide, with no difference between the 2 deep learning models. In other words, tweets labeled as “about suicide” are reliably actual tweets about suicide, and most such tweets are detected by the models. Thus, the use of any of these models for future research is very promising.

Using the final BERT models for both classification tasks, we estimated the percentage of tweets per category out of all suicide-related tweets in the United States from 2016 to 2018. Overall, approximately 6% were personal stories of concerned individuals, with approximately 5% on suicidal ideation or attempts and approximately 1% on coping stories. Estimates for awareness, prevention, and suicide case tweets were approximately 22%, 16%, and 16% of tweets, respectively. We plotted the daily volume per tweet category and investigated tweets during peaks in the time series. Most of these tweets were correctly classified by our models, and peaks often coincided with events matching a particular category (eg, the World Suicide Prevention Day or a celebrity suicide), which highlights the face validity of our model predictions. Finally, approximately three-fourths of all suicide-related tweets actually referred to someone taking their own life, whereas the rest used the term in another meaning or context (eg, euthanasia, suicide bombers, jokes, metaphors, and exaggerations).

### Limitations and Future Work

Despite our data set being more comprehensive than any existing data set on the topic, one of the limitations of our study is the size of the training data set, which is crucial for training deep learning models. In particular, this concerns the rarer categories that we have not yet used for machine learning in this study (eg, bereaved experiences and lives saved). The data set could further benefit from adding more examples of coping messages that describe suicidal ideation and behavior in the past, thereby implicitly indicating coping (see category definition in Multimedia Appendix 2). Furthermore, some tweets in the categories suicide other and off-topic might warrant to be investigated separately, given recent findings of the possible protective effects of flippant remarks and humor or negative portrayals of suicide in the form of murder-suicides [11]. Similarly, the suicide case category may warrant being separated into suicide news and condolence messages, which may have protective effects [9], and tweets about suicide cases may warrant filtering out those about celebrities [2]. Higher classification performance for the category suicidal ideation in the study by Burnap et al [24] showed that a focus on this category during data collection could improve our model.

Finally, a number of limitations apply to automated text analyses, such as machine learning. First, there are no traces of images, videos, or content of the URLs shared in the text of tweets, although this additional information can crucially affect the meaning of a tweet. Second, some things are only implicitly expressed or very subjective, and thus difficult to capture with such methods, but also difficult to reliably recognize for humans. For instance, it is difficult to clearly differentiate coping from suffering, even for humans who have some knowledge about how such experiences look like in the real world. It is even more difficult to capture such subjective experiences using word frequencies. Deep learning models such as BERT and XLNet, having been trained on huge amounts of text produced by humans, may be able to capture some of these nuances but require large amounts of training examples. Third, a machine learning model can only recognize example tweets that are sufficiently similar to the examples in the training set and only predict the predefined categories. In contrast, a human coder might recognize new ways of expressing the same meaning or the need to introduce a new category. We partially addressed the latter limitation through an extensive labeling process, ensuring that we captured all typical message categories by including a random set of tweets. Nonetheless, including more and different examples for suicidal ideation and coping stories in future studies would likely improve model performance.

### Conclusions and Practical Implications

The field of media and suicide research has only recently begun to evolve to consider social media content as relevant in the assessment of media effects. This study makes 2 major contributions to this field. First, it provides a systematic overview of different content types that are common on social media, which may be useful as a content labeling scheme for future research on the topic. Some of the categories identified have been found to be relevant to suicide prevention, particularly in other media types. For social media content, these associations with indicators of behavior, particularly suicidal behaviors and help-seeking, remain to be tested accordingly. Second, the machine learning methods enable researchers to assess large amounts of social media data and subsequently correlate it with available behavioral data of interest; for example, suicides or help-seeking data. In this way, this work enables systematic large-scale investigations of associations between these behaviors and fine-grained message characteristics of social media posts (eg, Niederkrotenthaler et al, unpublished data, May 2022; see Multimedia Appendix 1 for details). Such large-scale investigations will contribute to accumulating robust evidence on which characteristics are actually harmful and protective. Furthermore, future applications of the developed models might include the screening of social media content to detect other types of content associated with suicide cases that have not been described in previous research. The classification performances of the developed models demonstrate the strong potential of machine learning, particularly deep learning, for media suicide effects research.

## Appendix

Multimedia Appendix 1 Supplementary information with additional details on methods, results tables, search term lists, and word clouds.

Multimedia Appendix 2 Annotation scheme.

## Abbreviations

BERT: Bidirectional Encoder Representations from Transformers
LR: learning rate
SVM: support vector machine
TF-IDF: term frequency−inverse document frequency

## Data and Code Availability

The data sets generated and analyzed during this study are available on GitHub. The code and data for the statistical analyses and figures (intercoder reliability and frequency of categories) as well as CIs for machine learning are available on the web [47]. The code for training the machine learning models is available on the web [48]. Raw data only include tweet IDs, not tweet text, to protect the identity of the tweet authors. Tweets can be redownloaded via the Twitter API using these IDs.
