# Supplementary material for: Detecting Potentially Harmful and Protective Suicide-Related Content on Twitter: Machine Learning Approach
Source: J Med Internet Res. 2022 Aug 17;24(8):e34705. doi: 10.2196/34705 (PMC9434391; doi:10.2196/34705)

# Multimedia Appendix 1: Supplementary Information

# Detecting Potentially Harmful and Protective Suicide-related Content on Twitter: A Machine Learning Approach

**Authors**: Hannah Metzler[1,2,3,4,5], Hubert Baginski[3,6], Thomas Niederkrotenthaler[3], David Garcia[1,3,4]

**Affiliations:**

1. Section for Science of Complex Systems, Center for Medical Statistics, Informatics and Intelligent Systems, Medical University of Vienna, Austria
2. Unit Suicide Research & Mental Health Promotion, Department of Social and Preventive Medicine, Center for Public Health, Medical University of Vienna, Austria
3. Complexity Science Hub Vienna, Austria
4. Institute of Interactive Systems and Data Science, Graz University of Technology, Graz, Austria
5. Institute of Globally Distributed Open Research and Education, Austria
6. Institute of Information Systems Engineering, Vienna University of Technology, Vienna, Austria

## Follow-up study

The follow-up study of the current study that is repeatedly mentioned in the main manuscript was under review at the time of publication. The full reference is: Niederkrotenthaler, T., Tran, U. S., Baginski, H., Sinyor, M., Strauss, M. J., Sumner, S. A., Voracek, M., Till, B., Murphy, S., Gonzalez, F., Gould, M., Garcia, D., Draper, J., & Metzler, H. (under review). *Association of 7 million+ tweets featuring suicide-related content with daily calls to the Suicide Prevention Lifeline and with suicides, USA 2016-2018*.

## Dataset for Training Machine Learning Models

Time period: Because we initially started annotating tweets directly on the Crimson Hexagon platform, before downloading tweets for the target period (2013-2020), the training set includes a small fraction of 72 tweets from the years 2009 to 2012.

## List of search terms used to detect tweets for the training dataset

We started exploring the full dataset of tweets about suicide using keywords or phrases we suspected to be indicative of each of the five initial broad categories. After brainstorming some keywords (e.g. commit, lifeline, hope), we also used word frequency plots to identify typical terms in each category, and to search further example tweets with similar content. The list below includes all of these keywords, both those that worked well, as well as those that did not return many (too specific) or too many examples (too general). Asterisks indicate that all possible word endings were included, and terms in brackets indicate which examples we hoped to find with a keyword in cases where this is not obvious. We used no keywords for irrelevant tweets, given their high frequency.

**Suicide cases**

- Successful terms: commit*, found dead, news, heartbreaking, terrible, breaking news
- Too specific or general: police, jump*, hang*, hung, shot, shoot*, report*, suspect* (suspected suicide)

**Stories of coping**

- Successful terms: my story, my life, my experience, I (have) experienced, recover*, surviv*, strong, strength, thankful, okay, personal, I attempt*, my attempt, overwhelmed, fight, don’t want to die, grateful, despite, stay alive, suicidal thoughts
- Too specific or general: myself, thank, life, live, alive, try, story

**Prevention**

- Successful terms: lifeline, helpline suicide hotline, prevention, contact, help, support, protect, thoughts + struggling, alone, pain, anxiety, depression, warning signs, call, stay safe, stay alive, save + life (save someone’s life)
- Too specific or general: mental health, #mentalhealth, public health, first responders, #firstresponders, warning signs, call

**Awareness:**

- Successful terms: awareness, please retweet/copy, #mentalhealthawarenessmonth, #suicidepreventionweek, #suicideawareness, #nationalsuicidepreventionmonth,
- Too specific or general, or no notes on how successful they were: Risk, depressed, depression, anxiety, ptsd, loneliness, mental illness, trauma, homeless*, veterans, rape, sexual violence, message, talk, someone, feeling, upset, friends, reach out, stigma (#nostigma, #stopthestigma), struggles

## Text sequence length for model input

Figure S1 shows the distribution of tokens in the training set, based on which we determined the maximum sequence length for the models. The mean of the training tweet length is 25, the 95th percentile 57, and the 99th percentile 67 tokens. Therefore, we use a maximum sequence length of 80 tokens and cut off tokens beyond the maximum sequence length.

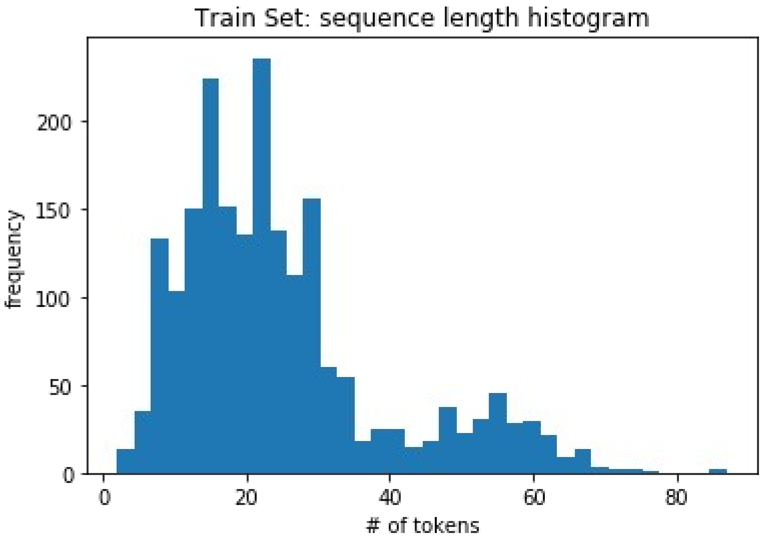


**Figure S1**. Histogram of the number of tokens (words etc) in the training set.

## Effect of different preprocessing strategies

For our main analyses, we only lowercase all words, and replaced URLs with http, and user mentions with @user. Additional NLP preprocessing best practises include removing digits, punctuation, stopwords, and lemmatization (grouping words according to their dictionary form). When working with longer texts, this eliminates frequent features that appear across most texts and provide little to no additional information. However, as tweets are already limited by a maximum content length of 280 characters, removing any information might worsen performance. We therefore explored how different preprocessing steps affect performance, first for lemmatization, and then for permutations of digit, stopword and punctuation removal. Lemmatization was not included in these permutations because punctuation and digits do not have lemmas, and the lemmas of stop words are often identical to the stop word (the, a , he, etc.).

We compare the different preprocessing strategies by training the base BERT model (12 layers, 12 attention heads, 110 million parameters, https://huggingface.co/bert-base-uncased) with a learning rate of 5e-5 for three epochs. Table S1 summarizes the results using the macroaveraged $F_1$-score and average accuracy across all classes. Removing stopwords, and applying lemmatization, respectively, always decreased model performance. Removing only digits (strategy 3) performs similarly to the basic preprocessing (strategy 1), with a slightly higher validation $F_1$ and slightly lower validation accuracy. Given that none of the additional preprocessing steps improved the performance, we apply strategy 1 for all analyses reported in the paper.

| | Preprocessing strategy | Validation set | | Test set | |
|---|---|---|---|---|---|
| | | ***$F_1$*** | ***Accuracy*** | ***F1*** | ***Accuracy*** |
| 1 | BERT-base (lowercase, http, @user) | 0.72 | 0.76 | 0.72 | 0.73 |
| 2 | + lemmatization | 0.69 | 0.75 | 0.70 | 0.73 |
| 3 | - digits | 0.73 | 0.76 | 0.71 | 0.74 |
| 4 | - punctuation | 0.72 | 0.76 | 0.71 | 0.73 |
| 5 | - stopwords | 0.65 | 0.71 | 0.64 | 0.69 |
| 6 | - digits - punctuation | 0.73 | 0.76 | 0.70 | 0.72 |
| 7 | - digits - stopwords | 0.66 | 0.72 | 0.65 | 0.7 |
| 8 | - punctuation - stopword | 0.65 | 0.71 | 0.65 | 0.70 |
| 9 | - digits -stopwords - punctuation | 0.65 | 0.71 | 0.66 | 0.70 |

**Table S1.** Macro averaged $F_1$ score and accuracy with different preprocessing strategies, training with a learning rate=5e-5 and 3 epochs.

## BERT and XLNet performance in 5 separate runs

The performance of BERT and XLNet, with fixed seeds and parameters, varies slightly from run to run owing to internal segmentation. We therefore ran these models 5 times, and report the results of five runs per task below. This illustrates that variance was generally low.

**Task 1: Six main categories**

| | Validation set | | | | Test set | | | |
|---|---|---|---|---|---|---|---|---|
| **BERT** | **Precision** | **Recall** | **$F_1$** | **Accuracy** | **Precision** | **Recall** | **$F_1$** | **Accuracy** |
| Run 1 | 0.75 | 0.74 | 0.74 | 0.78 | 0.73 | 0.70 | 0.71 | 0.74 |
| Run 2 | 0.72 | 0.72 | 0.71 | 0.75 | 0.71 | 0.71 | 0.71 | 0.74 |
| Run 3 | 0.71 | 0.71 | 0.71 | 0.75 | 0.71 | 0.70 | 0.70 | 0.73 |
| Run 4 | 0.74 | 0.69 | 0.71 | 0.76 | 0.69 | 0.65 | 0.66 | 0.71 |
| Run 5 | 0.73 | 0.68 | 0.70 | 0.75 | 0.74 | 0.67 | 0.70 | 0.73 |
| **XLNet** | **Precision** | **Recall** | **$F_1$** | **Accuracy** | **Precision** | **Recall** | **$F_1$** | **Accuracy** |
| Run 1 | 0.75 | 0.74 | 0.75 | 0.78 | 0.71 | 0.72 | 0.71 | 0.75 |
| Run 2 | 0.71 | 0.72 | 0.71 | 0.76 | 0.69 | 0.72 | 0.71 | 0.71 |
| Run 3 | 0.76 | 0.74 | 0.75 | 0.78 | 0.72 | 0.70 | 0.71 | 0.76 |
| Run 4 | 0.76 | 0.71 | 0.72 | 0.77 | 0.73 | 0.69 | 0.70 | 0.76 |
| Run 5 | 0.72 | 0.74 | 0.73 | 0.76 | 0.71 | 0.74 | 0.72 | 0.72 |

**Table S2. Macroaveraged performance metrics and accuracy across all 6 categories on the validation and test set.** Bold metrics are the best result per column. *Numbers in brackets indicate the learning rate, number of epochs, and the seed.

| Test set | Suicidal ideation & attempts | | | Coping | | | Awareness | | |
|---|---|---|---|---|---|---|---|---|---|
| **BERT** | **Precision** | **Recall** | **$F_1$** | **Precision** | **Precision** | **$F_1$** | **Precision** | **Precision** | **$F_1$** |
| Run 1 | 0.62 | 0.49 | 0.55 | 0.82 | 0.67 | 0.74 | 0.69 | 0.72 | 0.70 |
| Run 2 | 0.62 | 0.46 | 0.53 | 0.70 | 0.71 | 0.71 | 0.74 | 0.79 | 0.76 |
| Run 3 | 0.60 | 0.46 | 0.52 | 0.78 | 0.69 | 0.73 | 0.66 | 0.73 | 0.69 |
| Run 4 | 0.46 | 0.40 | 0.43 | 0.74 | 0.67 | 0.70 | 0.77 | 0.55 | 0.64 |
| Run 5 | 0.61 | 0.45 | 0.52 | 0.77 | 0.70 | 0.73 | 0.68 | 0.71 | 0.69 |
| **XLNet** | **Precision** | **Recall** | **$F_1$** | **Precision** | **Precision** | **$F_1$** | **Precision** | **Precision** | **$F_1$** |
| Run 1 | 0.49 | 0.70 | 0.58 | 0.69 | 0.69 | 0.69 | 0.71 | 0.72 | 0.71 |
| Run 2 | 0.57 | 0.60 | 0.58 | 0.67 | 0.79 | 0.73 | 0.65 | 0.72 | 0.69 |
| Run 3 | 0.66 | 0.44 | 0.53 | 0.72 | 0.69 | 0.71 | 0.70 | 0.81 | 0.75 |
| Run 4 | 0.69 | 0.39 | 0.49 | 0.74 | 0.74 | 0.74 | 0.72 | 0.69 | 0.71 |
| Run 5 | 0.59 | 0.56 | 0.58 | 0.74 | 0.81 | 0.77 | 0.67 | 0.78 | 0.72 |

| | Prevention | | | Suicide cases | | | Irrelevant | | |
|---|---|---|---|---|---|---|---|---|---|
| **BERT** | **Precision** | **Recall** | **$F_1$** | **Precision** | **Precision** | **$F_1$** | **Precision** | **Precision** | **$F_1$** |
| Run 1 | 0.84 | 0.87 | 0.85 | 0.75 | 0.77 | 0.76 | 0.64 | 0.71 | 0.68 |
| Run 2 | 0.83 | 0.87 | 0.85 | 0.78 | 0.78 | 0.78 | 0.62 | 0.65 | 0.64 |
| Run 3 | 0.82 | 0.88 | 0.85 | 0.75 | 0.74 | 0.75 | 0.61 | 0.68 | 0.65 |
| Run 4 | 0.75 | 0.92 | 0.83 | 0.73 | 0.81 | 0.77 | 0.69 | 0.54 | 0.61 |
| Run 5 | 0.81 | 0.89 | 0.85 | 0.74 | 0.75 | 0.75 | 0.62 | 0.67 | 0.65 |
| **XLNet** | **Precision** | **Recall** | **$F_1$** | **Precision** | **Precision** | **$F_1$** | **Precision** | **Precision** | **$F_1$** |
| Run 1 | 0.85 | 0.85 | 0.85 | 0.79 | 0.74 | 0.76 | 0.71 | 0.63 | 0.67 |
| Run 2 | 0.83 | 0.87 | 0.85 | 0.77 | 0.71 | 0.74 | 0.65 | 0.65 | 0.65 |
| Run 3 | 0.81 | 0.88 | 0.84 | 0.78 | 0.78 | 0.78 | 0.67 | 0.63 | 0.65 |
| Run 4 | 0.81 | 0.86 | 0.83 | 0.74 | 0.82 | 0.78 | 0.71 | 0.62 | 0.66 |
| Run 5 | 0.81 | 0.90 | 0.85 | 0.80 | 0.71 | 0.75 | 0.65 | 0.68 | 0.67 |

**Table S3. Intraclass performance metrics for deep learning models on the test set.**

**Task 2: About actual suicide**

| | Validation set | | | | Test set | | | |
|---|---|---|---|---|---|---|---|---|
| **BERT** | **Precision** | **Recall** | **$F_1$** | **Accuracy** | **Precision** | **Recall** | **$F_1$** | **Accuracy** |
| Run 1 | **0.86** | 0.80 | 0.82 | **0.88** | **0.85** | 0.81 | 0.83 | 0.88 |
| Run 2 | 0.89 | 0.80 | 0.83 | 0.89 | 0.87 | 0.78 | 0.81 | 0.88 |
| Run 3 | 0.83 | 0.81 | 0.82 | 0.87 | 0.87 | 0.83 | 0.85 | 0.89 |
| Run 4 | 0.83 | 0.81 | 0.82 | 0.87 | 0.85 | 0.83 | 0.84 | 0.89 |
| Run 5 | 0.84 | 0.86 | 0.85 | 0.88 | 0.81 | 0.82 | 0.82 | 0.86 |
| **XLNet** | **Precision** | **Recall** | **$F_1$** | **Accuracy** | **Precision** | **Recall** | **$F_1$** | **Accuracy** |
| Run 1 | 0.84 | 0.79 | 0.81 | 0.87 | 0.84 | 0.81 | 0.83 | 0.88 |
| Run 2 | 0.84 | 0.78 | 0.80 | 0.87 | 0.85 | 0.79 | 0.81 | 0.88 |
| Run 3 | 0.85 | 0.78 | 0.80 | 0.87 | 0.83 | 0.78 | 0.80 | 0.87 |
| Run 4 | 0.84 | 0.78 | 0.80 | 0.87 | 0.82 | 0.81 | 0.81 | 0.86 |
| Run 5 | 0.83 | 0.79 | 0.81 | 0.87 | 0.82 | 0.82 | 0.82 | 0.87 |

**Table S4. Macroaveraged performance metrics and accuracy for Task 2 (about suicide vs. off-topic) on the validation and test set.**

| **Test set** | **About suicide** | | | **Off-topic** | | |
|---|---|---|---|---|---|---|
| **BERT** | **Precision** | **Recall** | **$F_1$** | **Precision** | **Recall** | **$F_1$** |
| Run 1 | 0.90 | 0.95 | 0.92 | 0.80 | 0.67 | 0.73 |
| Run 2 | 0.88 | 0.97 | 0.92 | 0.85 | 0.59 | 0.70 |
| Run 3 | 0.92 | 0.95 | 0.93 | 0.82 | 0.72 | 0.76 |
| Run 4 | 0.91 | 0.94 | 0.93 | 0.80 | 0.71 | 0.75 |
| Run 5 | 0.91 | 0.90 | 0.91 | 0.72 | 0.73 | 0.73 |
| **XLNet** | **Precision** | **Recall** | **$F_1$** | **Precision** | **Recall** | **$F_1$** |
| Run 1 | 0.91 | 0.94 | 0.92 | 0.78 | 0.69 | 0.73 |
| Run 2 | 0.89 | 0.96 | 0.92 | 0.81 | 0.62 | 0.70 |
| Run 3 | 0.89 | 0.95 | 0.92 | 0.78 | 0.61 | 0.69 |
| Run 4 | 0.91 | 0.91 | 0.91 | 0.72 | 0.72 | 0.72 |
| Run 5 | 0.91 | 0.91 | 0.91 | 0.72 | 0.72 | 0.72 |

**Table S5. Intra-class performance metrics for Task 2 (about suicide vs. off-topic) on the test set.**

## Model vs. human performance

| Metric | Human-model | Suicidal | Coping | Awareness | Prevention | Suicide cases | Irrelevant |
|---|---|---|---|---|---|---|---|
| Precision | Coder 1 - BERT | 0.60 | 0.74 | 0.48 | 0.78 | 0.87 | 0.53 |
| | Coder 2 - BERT | 0.64 | 0.68 | 0.55 | 0.86 | 0.86 | 0.46 |
| | Coder 1 - Coder 2 | 0.82 | 0.82 | 0.71 | 0.90 | 0.82 | 0.52 |
| Recall | Coder 1 - BERT | 0.67 | 0.76 | 0.87 | 0.82 | 0.78 | 0.29 |
| | Coder 2 - BERT | 0.58 | 0.76 | 0.91 | 0.76 | 0.82 | 0.35 |
| | Coder 1 - Coder 2 | 0.67 | 0.88 | 0.64 | 0.75 | 0.87 | 0.70 |
| $F_1$ | Coder 1 - BERT | 0.63 | 0.75 | 0.62 | 0.80 | 0.82 | 0.38 |
| | Coder 2 - BERT | 0.61 | 0.72 | 0.69 | 0.81 | 0.84 | 0.40 |
| | Coder 1 - Coder 2 | 0.74 | 0.85 | 0.67 | 0.82 | 0.85 | 0.60 |

**Table S6. Inter-rater reliability per tweet category between two human coders and the BERT model, and between human coders in Task 2 in the reliability dataset.** The human coder listed before the hyphen in the column human-model was used as the ground truth. For each metric, the first two lines compare the model versus each coder, with the coder as the ground truth. The third line reports the same metrics for coder 2 compared to coder 1 as the ground truth.

## Word clouds for all tweets in categories about actual suicide

All below word clouds are based on tweets in the entire labelled dataset (including the train- and test set).

### Suicidal ideation and behavior

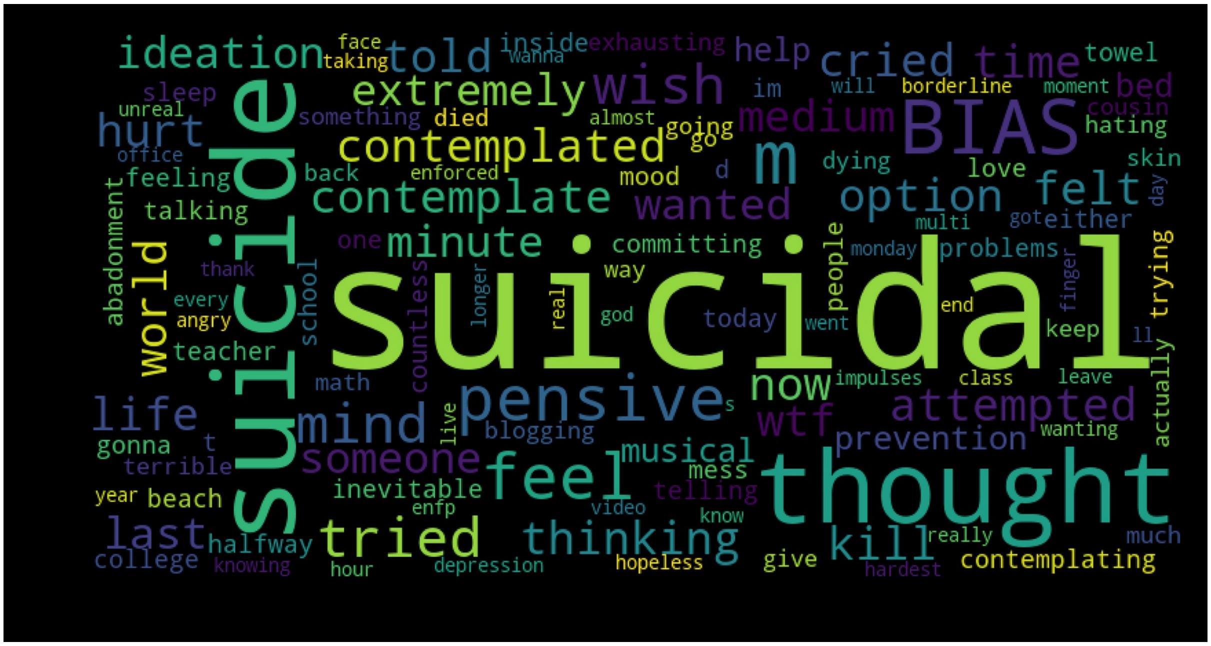


### Coping

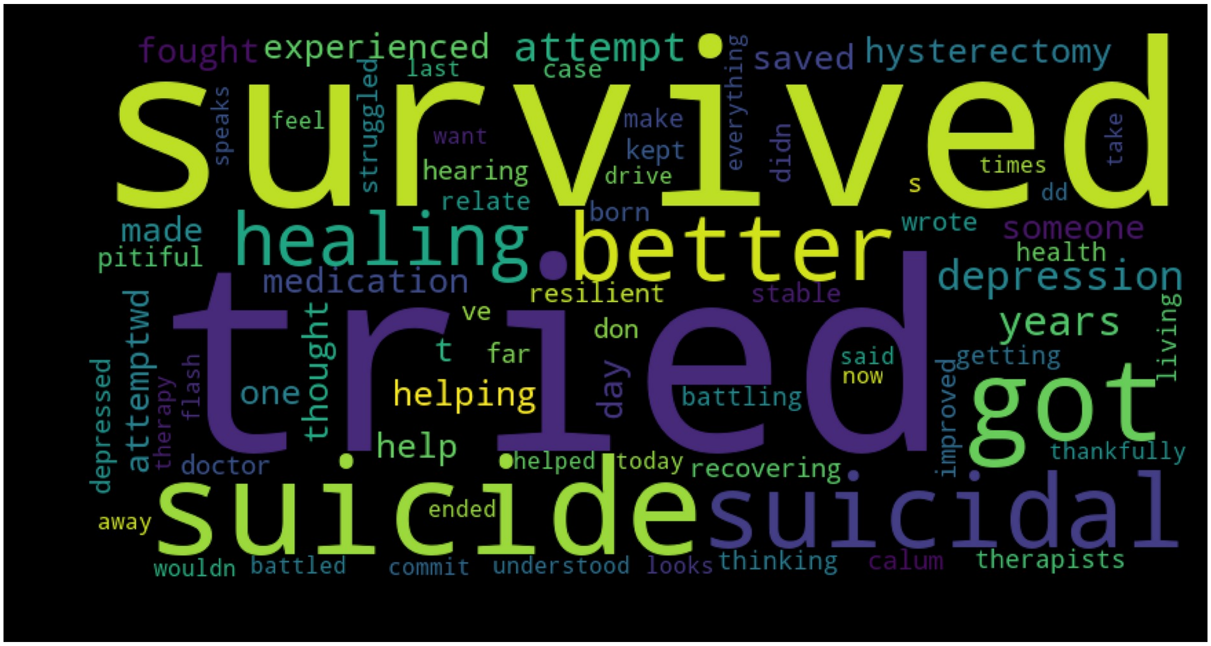

**Prevention**

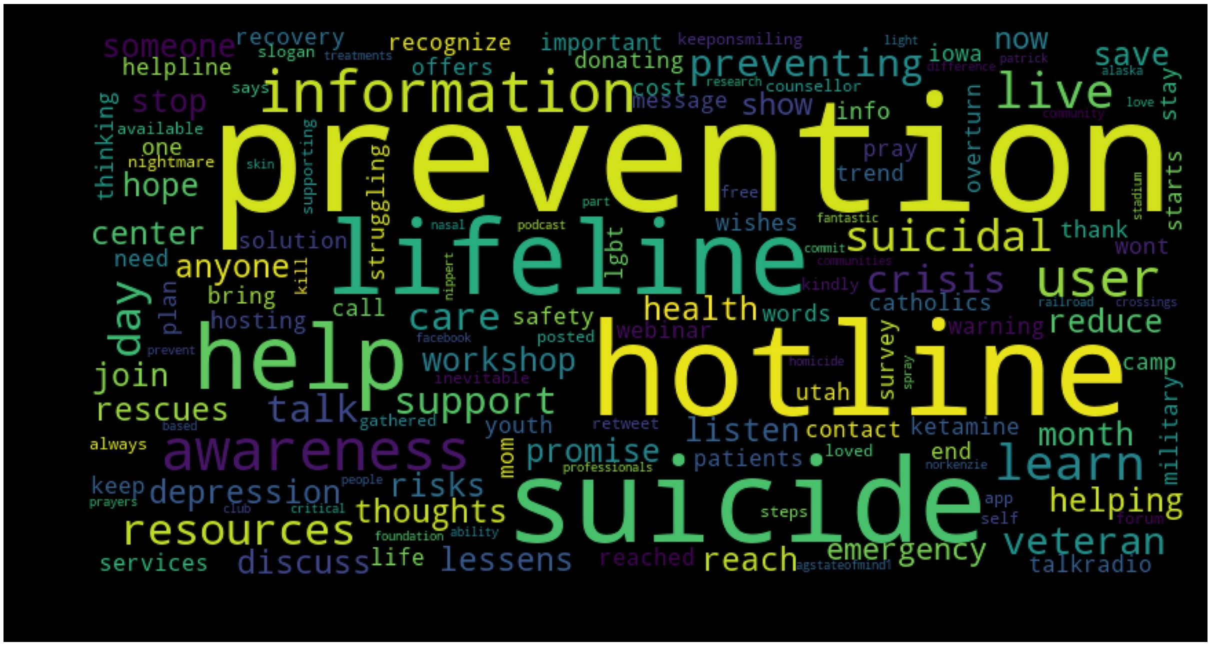


**Awareness**

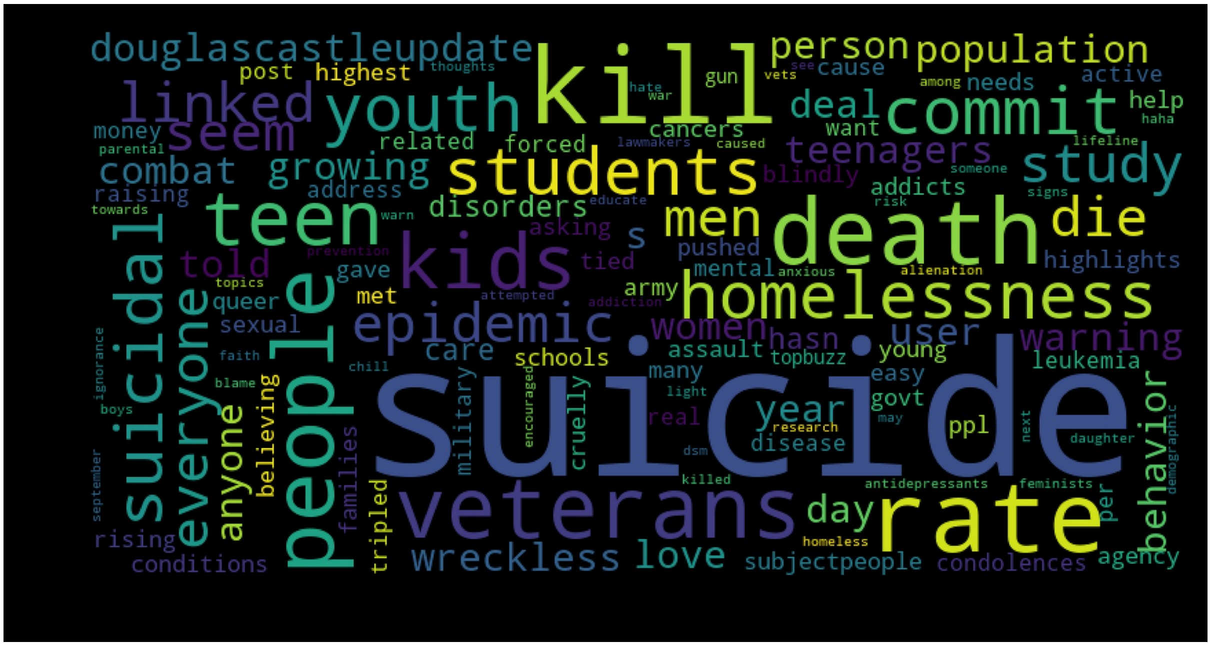

**Suicide cases**

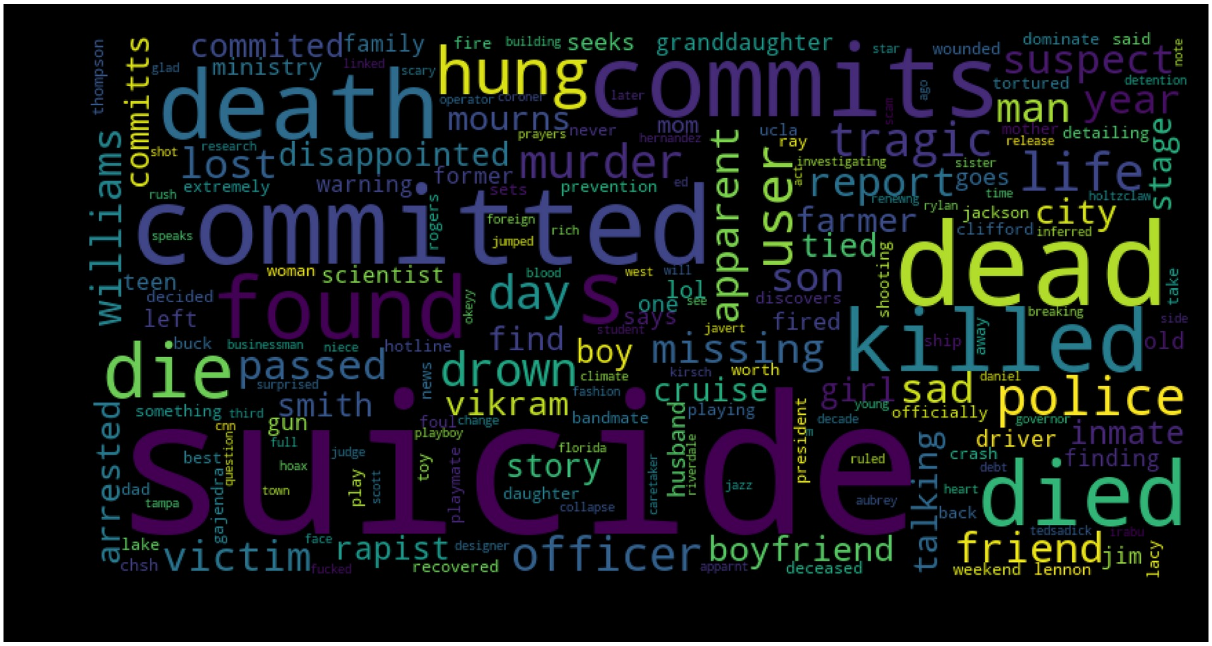


**Suicide other**

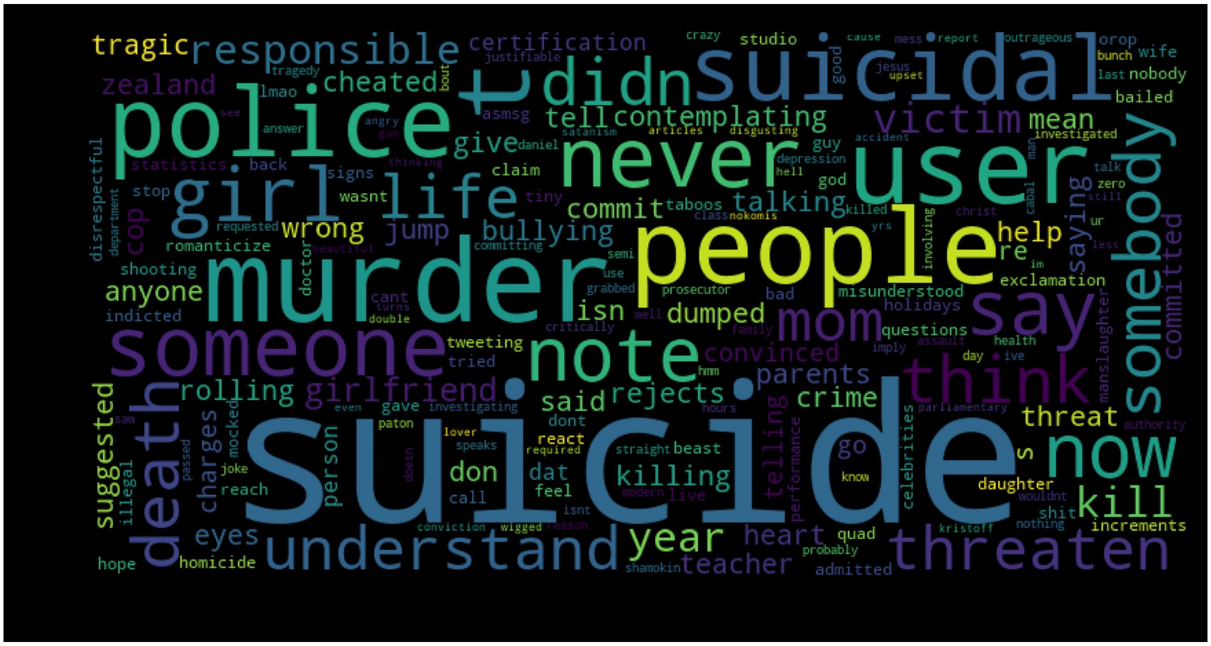

**Bereaved negative**

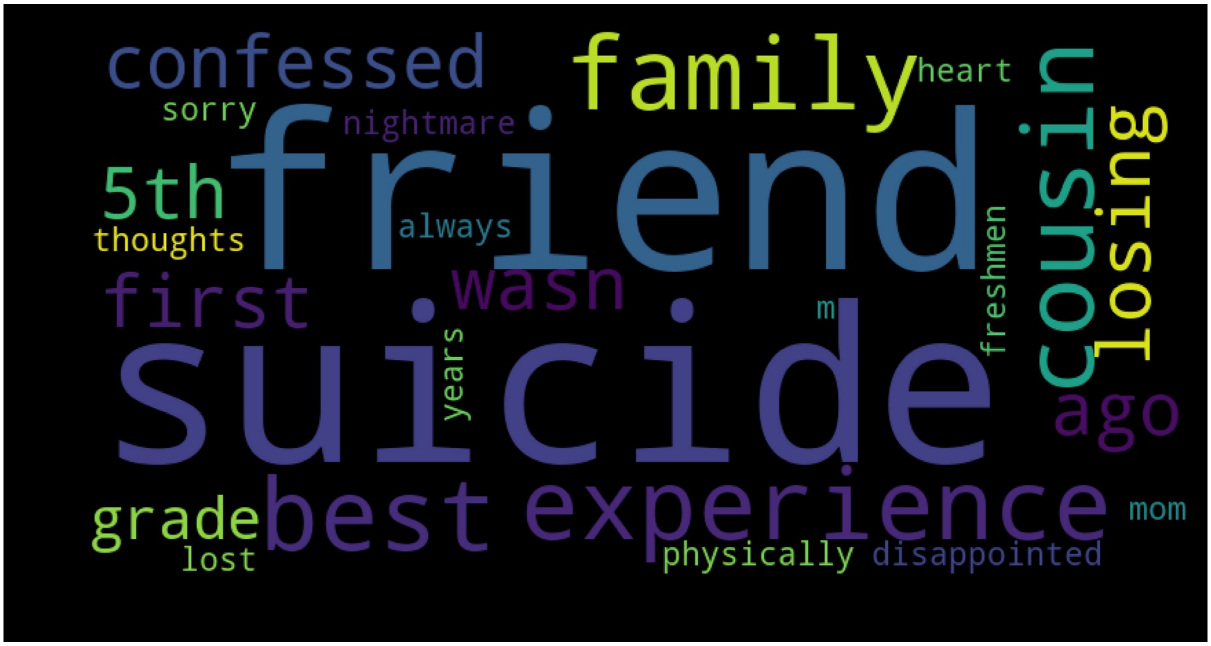


**Bereaved coping**

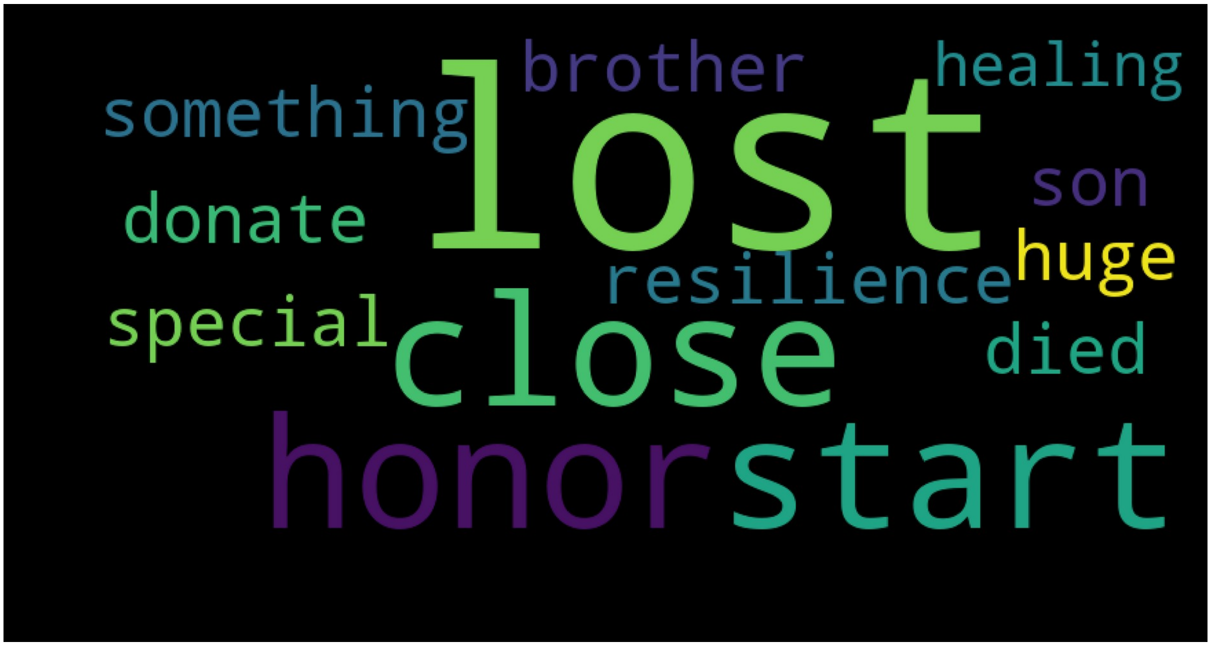

**News suicidal ideation and attempts**

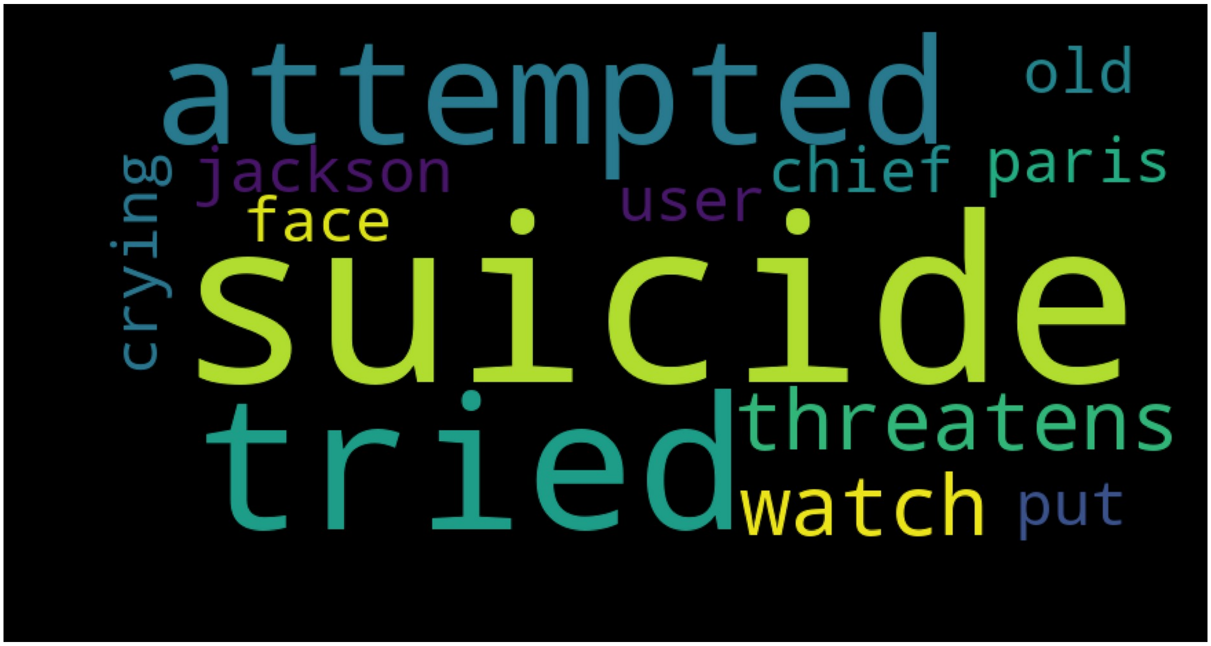


**News coping**

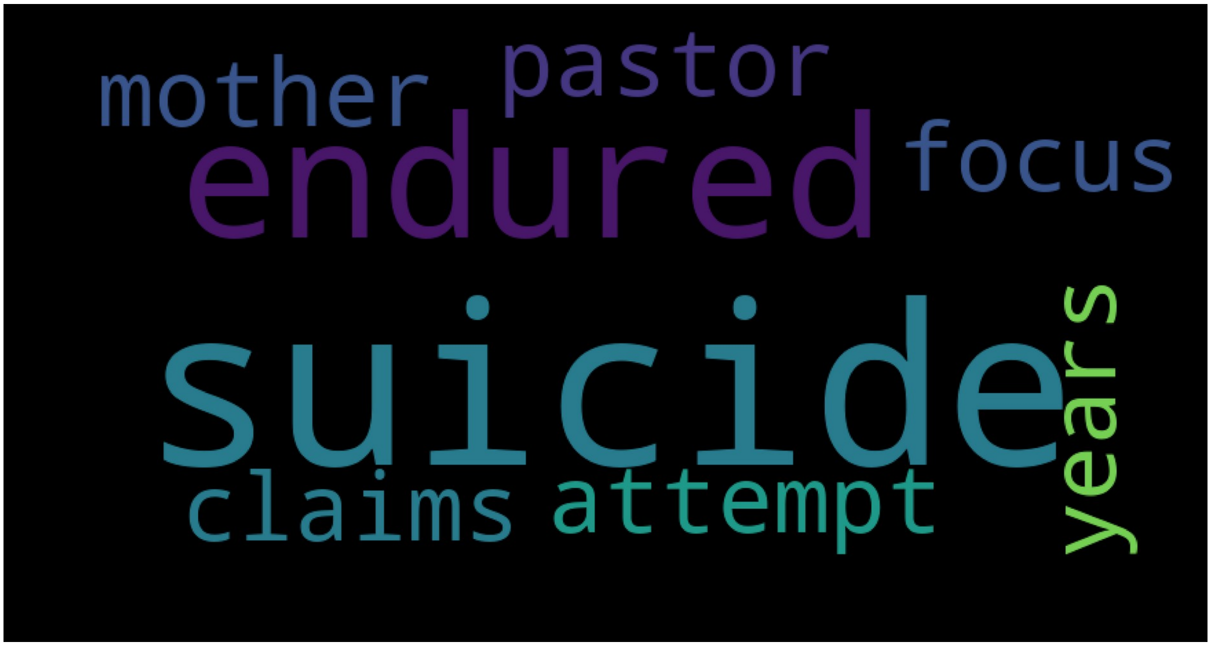

Supplement: Multimedia Appendix 1 [file jmir_v24i8e34705_app1.pdf]
