# Supplementary material for: Detecting Potentially Harmful and Protective Suicide-Related Content on Twitter: Machine Learning Approach
Source: J Med Internet Res. 2022 Aug 17;24(8):e34705. doi: 10.2196/34705 (PMC9434391; doi:10.2196/34705)
Supplement: Multimedia Appendix 2 [file jmir_v24i8e34705_app2.pdf]

Multimedia Appendix 2

Annotation scheme with detailed category definitions and tweet examples

Authors: Hannah Metzler[1,2,3,4,5], Hubert Baginski[3,6], Thomas Niederkrotenthaler[3], David Garcia[1,3,4]

Affiliations:

1. Section for Science of Complex Systems, Center for Medical Statistics, Informatics and Intelligent Systems, Medical University of Vienna, Austria
2. Unit Suicide Research & Mental Health Promotion, Department of Social and Preventive Medicine, Center for Public Health, Medical University of Vienna, Austria
3. Complexity Science Hub Vienna, Austria
4. Institute of Interactive Systems and Data Science, Graz University of Technology, Graz, Austria
5. Institute of Globally Distributed Open Research and Education, Austria
6. Institute of Information Systems Engineering, Vienna University of Technology, Vienna, Austria

Overview of dimensions and categories

Tweet categories are organized along two dimensions, the type of message and the underlying perspective with regard to solutions and coping. The combination of these two dimensions divides postings into 10 categories of interest, and 2 irrelevant categories. A third dimension applies only to irrelevant tweets, and indicates if a tweet is *serious or not serious*. Dimensions and categories are described below, followed by detailed instructions and examples for tweet annotation.

		Underlying perspective	
**Message type**		**Problem & Suffering**	**Solution & Coping**
**Personal experiences** 1st or 3rd person		Suicidal ideation & attempts	Coping (Papageno)
**News about experiences & behavior**		News suicidal ideation & attempts	News coping
**Experience of bereaved**		Bereaved negative	Bereaved coping
**Case reports**		Suicide cases (Werther)	Lives saved
**Calls for action**		Awareness	Prevention
**Irrelevant**	**Suicide other**	Murder-suicides, history, fiction, not being suicidal, opinions...	
	**Off-topic**	Bombings, euthanasia, jokes, metaphors, band/song names...	

Dimensions

Dimension 1: Message Type

We distinguished between six types of messages:

1. Personal stories about experiences: Experience of an affected individual either in first or third person perspective
2. News reports about suicidal experiences and behavior except cases, often about celebrities
3. Personal experiences of bereaved individuals: Describes the experience of a person who lost someone to suicide in first or third person perspective, including news reports.
4. Reports of particular completed or prevented suicide cases, often news reports
5. Calls for action: These are general statements calling for actions addressing the problem of suicide, and intending to spread problem awareness or prevention-related information.
6. Irrelevant: Message does not fit into any of the above categories. Here we distinguished between messages that were about suicide but did not fit in the above categories (suicide other), and those that were entirely off-topic (i.e. not about someone taking their life).

Dimension 2: Underlying Perspective

Perspective refers to whether the tweet seems to communicate that there are hope and solutions for dealing with, and/ or potentially preventing, suicide. This may be expressed implicitly or explicitly. We distinguished:

1. Messages that frame suicide only as a problem and/or from an exclusively negative/suffering perspective.
2. Messages implying that solutions and ways of dealing with the problem exist, in a neutral or positive tone, but not negative. Any hint at a solution, alternative strategies, an attempt of coping, ways of becoming active or supporting efforts to fight suicide count.
3. Messages that do neither (only possible for messages in the irrelevant category)

Tweets in the category *suicide other* could be labeled as either of those as well as neither, and off-topic tweets were all labeled as neither, given that they focus on another topic.

Dimension 3: Serious vs. Not Serious

Only tweets that were clearly about suicide in the sense of someone's life ending counted as serious, whereas metaphors, exaggerations, sarcasm and jokes counted as not serious. If it was unclear whether a tweet was serious (e.g., sarcastic tweets, exaggerations), it was labeled as not serious. Only tweets in the two irrelevant categories could be labeled as not serious, given that all others are about actual suicide.

Categories

- ***Suicidal ideation & attempts:*** Personal stories about an individuals negative experience with suicidal thoughts, related suffering (e.g. depression), suicidal communication and announcements, or suicide attempts, from the perspective of an affected individual, in 1st or 3rd person perspective.
- ***Coping***: Personal stories about an individuals experience with suicidal thoughts or a suicide attempt, with a sense of hope, recovery, coping, or mentioning an alternative to suicide. The sentiment does not have to be positive. A neutral tone, or talking about difficult experiences with a sense of coping or mentioning recovery, is sufficient.Previous research suggests such messages may have a *Papageno effect.*
- ***News suicidal ideation & attempts:*** News reports about suicidal experiences without any mention of coping, including reports on suicidal ideation, suicide attempts, announcements of suicide, someone being put on "suicide watch", etc., often about celebrities
- **News coping:** News reports about attempted or successful coping with or recovering from a suicidal crises, often about celebrities
- ***Bereaved negative:*** Describes the suffering or purely negative experience of a person who lost someone to suicide, including depression, grief, loss etc. These tweets necessarily refer to a suicide case, but are labeled as bereaved as long as they focus on the experience of bereaved individuals.
- ***Bereaved coping:*** Describes the experience of a bereaved person with a sense of hope, recovery or coping. The sentiment does not have to be positive. A neutral tone, or talking about difficult experiences with a sense of coping or mentioning recovery, is sufficient.
- ***Suicide cases:*** About an individual suicide, or a timely or geographical suicide cluster. Suicide cases have priority over definition criteria of other categories (except tweets focusing on bereaved individuals which are always related to a suicide case). Previous research suggests such messages on individual suicide deaths (especially about celebrities) may have a *Werther effect*.
- ***Lives saved:*** News report or personal message about someone saving a life. In contrast to prevention tweets, these lives are often being saved coincidentally.
- ***Awareness:*** Tweets intending to spread awareness for the problem of suicide, often focusing on high suicide rates or associations with bullying, racism, depression, veterans etc. without hinting at any solution. These are often reports of research findings or suicide statistics. Mere expressions of intent to help count as awareness, but any hint at something that can be done counts as prevention.
- ***Prevention:*** Tweets spreading information about a solution or an attempt to solve the problem of suicide, including prevention at an individual (e.g. do not leave people alone in crisis situations) or public health level (e.g. safety nets on bridges). Hinting at a solution or a way of dealing with the problem is enough. No specific action needs to be described. These tweets often include a helpline number. Announcements of prevention events and broad recommendations for actions also count: donations, prayers with a focus on a solution for suicide, being there for someone, telling people that they matter, taking a course about suicide prevention, warning signs to watch out for,
- ***Suicide other:*** Anything about suicide but not clearly related to any other above category, including murder-suicides, confident statements that something was not a suicide, convincing

statements of not being suicidal, historical tweets about suicides that are a minimum of 40 ago (e.g. about Hitler's suicide), movies, books, novels, fiction about suicide

- ***Off-topic:*** all messages that use the term suicide in a context other than someone taking their own life due to suicidal thoughts. This includes messages on euthanasia, suicide bombing, and suicide attacks, messages that are (suspected) jokes, irony, sarcasm, flippant remarks, or really unclear in terms of authenticity, messages that use suicidal/suicide to exaggerate an emotional experience (unclear if serious), or as a methaphor (e.g. political/financial/career suicide, suicide workout, suicidal immigration policies...), and messages about "suicidal animals" (e.g. killed by car).

For cases of tweets that cannot be clearly assigned to only one category, the instructions below include prioritization rules. These are based on:

- Strength evidence for this content type being associated with suicide rates from previous research (e.g., We have stronger evidence that suicide case reports may have harmful effects on suicide rates compared to awareness tweets, so if an awareness tweets includes a case report, this is labeled as suicide case.)
- Logical necessity (e.g., experiences of bereaved necessarily include a suicide case, but are only labeled as suicide case if they focus on the case, rather than the experience of the bereaved individual)
- The underlying perspective of the tweet: coping/solution oriented tweets might still mention a problem and suffering, since coping with suicide includes dealing with negative experiences. We therefore generally prioritize the coping/solution oriented category over the problem-centered category within the same message type when both elements are present (e.g., within personal stories on suicide, coping stories were prioritized over suicidal ideation stories).

Detailed instructions for annotating tweets

The below tweet examples, if not published by news agencies, have been anonymised by replacing some words with synonyms, changing punctuation and deleting user names and links, so that the user's who published them on Twitter cannot be identified.

1) Personal experiences of affected individuals

- Experience of an affected individual either in first or third person perspective, indicated by Coping1/Suicidality1 vs. Coping3/Suicidality3
- Whenever a personal experience is mentioned, this has priority over awareness- or prevention-related statements in the same tweet. We code as personal or bereaved experience.
- If 1st and 3rd person perspectives are mixed, in a tweet from a third person perspective that quotes a 1st person perspective, code as 1st person.
- These personal experience categories do not apply in the following cases:
  - General statements on suicidal thoughts do not qualify
  - Personal experience of bereaved individuals go in a separate category
  - Any thought that is really unclear in terms of authenticity should go under off-topic (sarcastic, using suicidal to exaggerate an emotional experience)
  - If they seem posted by news outlets, they go into category 5.2: Coping news

1.1) Suicidal stories: Stories of suicidal ideation and attempts

- About individual suicidal thoughts or suicide attempt from the perspective of an affected individual, negative experiences and thoughts, suffering, depression, …
- Suicidal communication/announcements of suicide

Examples

- Suicidality1: It really fucking pissed me off because I am depressed and suicidal, but she of course doesn't know that.
- Suicidality1: My mother thinks that I am suicidal. She's not wrong.
- Suicidality1: Suicidal Thoughts.I will pretend things are okay as I always do.
- Suicidality3: My best friend just left the hospital after another suicide attempt. I knew something bad had happened...
- Example for exaggerations, where it is not clear if the writer is actually suicidal or making a joke/using suicide as a metaphor for feeling really bad:
    - The way I feel I want to commit suicide right fucking now!
    - My suicide note will be a math worksheet. I can't do it anymore
    - These people make me go suicidal.

1.2) Coping stories: Stories of hope, recovery, and coping

- About an individuals experience with suicidal thoughts or a suicide attempt, with a sense of hope, recovery, coping, or mentionning an alternative to suicide.
- Does not have to be positive, but cannot be negative, neutral is enough. If not clearly negative, priority is on coping. These tweets could have a Papageno effect.
- Tweets that clearly indicate the experience of suicidal ideation or behavior lies in the past also belong here, because they suggest the person survived.

Examples

- Coping1: I realized that I did not want to die. I just did not want to continue my life as I knew it.
- Coping1: I simply don't feel valued. Sometimes, the ONLY thing that kept me from suicide was my dad and my love for myself that i had to find.
- Coping3: I have talked someone out of suicide, it is not that fucking scary.
- Coping 1 - expressing coping implicitly by talking about suicidal ideation or attempt in the past:
    - Back on November 5th I tried to commit suicide. I had no intent to continue living.
    - I was suicidal in the 6th and the 11th grade.

2) Experience of bereaved individuals

- Describes the experience of someone losing a close one to suicide from a first or third person perspective of bereaved individuals, including news reports about bereaved individuals.
- Simply mentioning someone’s suicide is not sufficient, the tweet needs to describe or implicitly hint at the bereaved person’s emotional experience related to the suicide.
- General statements (e.g., RIP followed by the name of a deceased person) don‘t qualify.
- These tweets necessarily refer to a suicide case, but are only labeled as suicide case rather than experience of bereaved when focusing on the suicide rather than the experience of bereavement.

2.1) Bereaved negative: negative experience, suffering

- About a bereaved individuals negative experience/suffering with the suicide of a close one. Suffering, Depression, desperation, grief, loss, without a sense of coping or hope.
- If a tweet includes both coping/improvement and suffering, label it as coping.

Examples

- I am very sensitive about suicidal comments like "kill yourself". My best friend did this. I’ll kick your ass.

- The little brother of my best friend just attempted suicide. I’m about to start crying. Oh my god.
- Implicitly hints at suffering: 4 years ago my ex-girlfriend committed suicide. [Loudly crying face] God please make sure she is okay. :'([folded hands]

2.2) Bereaved coping: experience of coping, hope, recovery

- About a bereaved individuals experience with the suicide of a close one, with a sense of hope, recovery, and coping, every attempt of coping counts (outcome need not be positive)
- Does not have to be positive, but cannot be negative, neutral is enough. If not exclusively negative, label it as coping.

Examples

- My girlfriend killed herself 12 years ago. Griefing myself, I watched others devastated by grief fall into a dark hole and never recover. So I chose to open my heart, deepen compassion and meditation, and go toward service. These were the gifts of grief. My life is better for all of it.
- The actor opened up about his mother's scary suicide attempt and how he quickly saved her from dying.
- Everyone has a story. After suicide of a friend, I wrote #OnPoint. Let's also write your story together!

Examples that do not qualify because they do not describe the bereaved person’s experience:

- My mom committed suicide 20 years ago. He had PTSD and was a Vietnam veteran, and this is really really important.
- My brother killed himself 7 years ago. I just found out I’m the beneficiary of his life insurance. What should I know?

3) Suicide cases

3.1) Suicide cases (Werther)

- About an individual suicide, or a suicide cluster (timely or geographical)
- These tweets could have a Werther effect.
- Suicide cases have priority over definition criteria of other categories:
  - If it is about prevention or awareness but still obviously related to a suicide or attempt, code here. Even if lifeline is mentioned or a solution focus is present, if a suicide case is tweeted, it belongs here. This means Werther tweets can very rarely have a solution focus, i.e. receive the label 2 in the column focus.
  - Only bereaved stories about a suicide with a clear focus on personal experience have priority over Werther. Personal stories about attempts belong into suicidality1/3.
  - If doubts are expressed if a death was a suicide , we still code it as a suicide case. E.g. „apparent suicide“, „possible suicide“, „suspected suicide“. (An individual at risk would perceive it as a suicide case.)
- Suicide by cop also qualifies
- Suicides of villains or criminals also qualifies

Examples:

- Staten Island boy commits suicide after bullying ignored @TwURL
- Married man attacks TN college girl, commits suicide: A married man who… @TwURL #SuryaRay #India
- It is a beautiful cliff. Many people commit suicide here.

- Villain: Steuben County man accused of murder dies by suicide.
- Suicide by cop: Utah police: Man killed by officers in 2014 intended to 'commit suicide by cop'
- Prevention-related but mentioning a suicide case, therfore code here: What a tremendous loss with the passing of Kate Spade. My condolences to her family and loved ones. Depression does not discriminate. National Suicide Prevention Lifeline: 1-800-273-8255
- Expressions of doubt:
  - Death of Lennon Lacy ruled a suicide by the FBI, questions still linger
  - Former Patriot Aaron Hernandez found dead in prison in apparent suicide

3.2) Life saved

- News reports about someone saving a life, opposite of Werther tweets
- In contrast to prevention tweets, these lives are often being saved incidentally.

Examples

- Authorities: Kayaker rescues man who jumped from bridge: Authorities say a man who attempted suicide by jumpin...
- All the commotion up by Callery Park is LPD trying to help a suicidal 16-year-old girl. She's safe now, but sounds like she's in bad shape
- thedailybeast: Dolphins saved a girl from committing suicide. Susan Casey explains how it happened here: …
- #MLB Wire: How a former Cowboys player helped stop John Daly from committing suicide

4) Calls for action

- Calls for action: These are general statements calling for actions addressing the problem of suicide, intending to spread awareness or prevention-related information.
- If a personal story is told (affected individual or bereaved), the tweet does NOT count as awareness or prevention, even if it includes other content typical for these categories.
- If the below rules are not sufficient to distinguish between awareness and prevention tweets, because the tweet contains both elements, judge what the focus of the message is (focused on problem/suffering vs. solution). See the examples for distinguishing these two categories below.

4.1) Awareness

- Tweets focused on the problem of suicide and high suicide rates, or associations with bullying, racism, depression, veterans etc. without hinting at any solution.
- These tweets might want to help prevent but give no recommendation on what to do and how.
- Often includes research findings that do not explicitely say what an individual can do to prevent suicide. These research findings do not need to be written with the intention to help – pure information/updates are awareness tweets too.
- Often these tweets ask other Twitter users to retweet information. Mere retweeting does not count as a solution, because it does not hint at anything that can be done to prevent suicide. Therefore, these are awareness rather than prevention tweets.

Examples Awareness:

- Suicide risk linked to insomnia, alcohol use, study shows: Insomnia symptoms mediate the relationship between ... @TwURL
- Kinda sad & creepy. Suicide forest, Japan. 200 people attempt suicide in this forest every year.

- EverydayHealth: ABC News Chief Medical Correspondent drjashton's new book shines a much-needed spotlight on the pain and struggles faced by people who have lost a loved one to death by suicide @TwURL
- Reblog if you would be devastated if you found out one of your followers committed suicide. - iiamylou:... @TwURL
- Research finding: Nearly 78 percent of the 45,000 people who kill themselves every year in the United States are men. Here’s what the research on masculinity tell us about this crisis: @TwURL
- 22 veterans commit suicide every day. Would at least 22 of my Twitter friends please copy this and retweet?

4.2) Prevention

- Tweet is on prevention on individual level (e.g. do not leave people alone in crisis situation) or public health level (e.g. safety nets on bridge)
- Focused on a solution or an attempt of solving the problem. Hinting at a solution or a way of dealing with the problem is enough. No specific action needs to be described.
- Tweets often have a help-line in focus. Mentioning the helpline is sufficient for indicating a first step towards a potential solution.
- General broad recommendation actions go here as well: donations, prayers with a focus on a solution for suicide, being there for someone, telling people that they matter, „call me“ without providing a phone number, taking a course/class/seminar about suicide prevention, warning signs to watch out for, announcing/advertising a prevention event

Examples Prevention:

- Individual prevention: When someone is suicidal you need to be there i don’t care what’s going on, drop what you are doing and be there for them.
- Individual and helpline: @SpringByrum RE: CluelessMomTho1. Please recommend her to call Suicide Prevention. It’s disturbing that people think it’s so taboo, they will not speak of it. The number is toll free and anonymous. God bless and have a blessed New Year.
- Helpline: You are loved, you are heard, you are not alone. Suicide Prevention 800-273-8255 Mental Health Hotline 888-991-4284 Center Against Sexual Assault 866-373-8300 National Alliance on Mental Illness 800-950-6264 National Sexual Assault 800-656-4673
- Helpchat: Chat - Idaho Suicide Prevention Hotline @TwURL
- Rather negative, but mentions helpline: #MensHealth is #MentalHealth. ‘74% of persons who died by suicide in Austin (2013-2017) were men - City of #Austin National Suicide Prevention Lifeline: 1-800-273-8255 City of Austin: Insured / Uninsured, visit @TwURL 24/7 Helpline 512-472-HELP (4357) @TwURL
- Unspecific, but solution-focused: 100 reasons not to commit suicide
- Event: #Seminar 07-17: Disarming the Suicidal Mind #BehavioralHealth #MO #StLouis @TwURL
- Public health level: Golden Gate Bridge suicide barrier funding approved - CBS News @TwURL
- Prevention news: Some Nebraska businesses are taking a creative approach to raise awareness around this issue, while also raising money to support local organizations that focus on veteran and service member suicide prevention. @TwURL

Examples to distinguish Awareness vs. Prevention:

- Solution focus that is totally irrelevant to suicide prevention, therefore awareness: Day 53 of 22 push-ups a day for infinite days. Spread the word that Veteran suicide is not the answer. #22PUSHUPS for #22KILL @TwURL
- Attempt at helping, no problem focus, therefore prevention: To everyone who has dealt with suicidal thoughts this past year: I'm glad you're still here :)

- Superficial solution, but not problem-oriented, therefore prevention: Suicide is a permanent solution to a temporary problem. Always remember that you are loved and NEVER alone.
- Prevention because more hope than problem focus: I just pray that everyone that has suicidal thoughts realizes that there is always a path to happiness. OR: SPECIAL PRAYER FOR THOSE WITH SUICIDAL THOUGHTS: Fear thou not
- Prevention, because there is a focus on listening, which is equivalent to offering a solution. Without the comment about listening, this would be awareness: Would three of my Facebook friends please copy and repost. I'm doing this to prove that someone is always listening. #SuicideAwareness
- Awareness, because it focuses on a problem (ambiguous case because it possibly hints that social inclusion on TV should be improved, but it does so by pointing out the problem, rather than saying “let’s improve social inclusion”): @User 90% of Indigenous Australians deaths by suicide are under 30 year of age. Social inclusion on our TV matters.
- Awareness, because it only includes the word prevention, but does not hint at a solution: The 2nd leading cause of death in the US for ages 10-34 is suicide. Please reach out. #SuicidePrevention #theyellowelephant #oktobenotok #Mentalhealth @TwURL
- Awareness, because no solution, only retweeting (which does not prevent suicide): “@User: Every 17 seconds someone commits suicide because of cyber bullying. RT if you're against cyber bullying.”

5) News about suicide (except suicide cases)

5.1) News about suicidal ideation and attempts

- News reports about suicidal ideation and attempts, often about celebrities, without any mention of coping
- Tweets (3rd person perspective) that mention „threat of suicide“, or someone being put on suicide watch:

Examples

- “Threat of suicide“ , or suicide watch:
- ENDANGERED EAGLE? Ex-rocker put on suicide watch after wife’s death: @TwURL
- Vine Star Threatens Suicide After Video of Him Pressuring 16-Year Old Girl Into Oral Sex S… @TwURL
- Ray J's Girlfriend -- I Made a Mistake ... Suicide Threat Was Wrong: Ray J's girlfriend says she'd never kill … @TwURL
- Scarface @User speaks on suicidal thoughts @TwURL
- Iggy Azalea reveals she contemplated suicide @TwURL

5.2) News about coping and coping attempts

- News reports about coping with suicidal crises, recovery, attempts in the past etc., usually about celebrities
- Even if quotes from the first person perspective, code tweets as Coping_news rather than Coping1 because of their reach.
- Tweets about past suicidal experiences, because they suggest the person coped with the crisis.

Examples

- Gisele Bündchen Says She Battled Panic Attacks So Extreme She Considered Suicide: 'I Felt Powerless' Celebrities›Interviews The supermodel opens up about her battle with panic attacks. She’s one of the highest-paid supermodels in the world, marrie… @TwURL
- Demi Lovato: I Was Suicidal at Age 7 @TwURL #knssradio

- Fox News Holly Madison's book reveals she contemplated suicide Fox News "Peepshow" star and former Playboy… @TwURL #Playboy

0) Irrelevant

0.1) Suicide other: about suicide but not in our categories of interest

- Anything about suicide but not clearly related to any other category goes here
- Murder-suicides
- Statements that something was not a suicide, with relatively high confidence.
- Someone convincingly saying they are not suicidal
- Personal opinions, e.g. people saying they don't understand why people make jokes or laugh about suicide
- Historical tweets about suicides that are long ago (e.g. about Hitler), minimum 40 years (i.e. longer than Kurt Cobain, who might still have an influence)
- Movies, books, novels, fiction about suicide

Examples

- 12 Surprising Things That Happened On The Ground Immediately After Hitler Killed Himself @TwURL
- It's a response about anyone who has committed suicide, for any reason. Suicide is a personal choice, it's never the fault of anyone else. @TwURL
- Am I just thinking this is weird or does it seem like all teens are considering suicide?
- Fiction: I got more pussy after she killed herself than when she was my wife. This movie is fucked up.

0.2) Off-topic: not related to topic of actual suicide

- Jokes, suspected irony or sarcasm, flippant remark
- Using „ suicidal/suicide“ to exagerate an emotional experience (unclear if serious), suspected exaggeration
- Suicide and suicidal as methaphors (e.g. political/financial/career suicide, suicide workout, suicidal immigration policies...)
- Any thought that is really unclear in terms of authenticity goes here.
- Euthanasia, suicide bombing, suicide attacks, tweets are off-topic
- Tweets about “suicidal animals”, e.g. killed by car
- Animal suicides (see examples)

Examples

- You'll commit suicide trying to read my mind
- [HELP] last try until I take the suicide plunge to 10.5 on my iPhone 6 @TwURL
- Metaphors:
    - After 11 hours of meetings and about 3 hours in the car on suicidal roads, I realised it's January.... now i'm depressed
    - "@User: Even hearing your name makes me want to commit suicide. I know the feeling!
    - Before you join the lemming line of b2b marketers flocking to all social: 15 Things to Consider Before You Commit Print Suicide @TwURL
    - He is commiting career suicide
    - Our country is committing policital suicide.

- Exaggeration:
  - After having the nail polish come off one of my nails I'm basically contemplating suicide
  - I forgot how suicidal math classes make me.
  - So I'm laying on my floor and I looked up to see this and almost committed suicide @TwURL
- Animal suicides:
  - A suicidal bird flew into my car.
  - I think my dog is suicidal.
  - Damn raccoon must have been suicidal, walking so slowly across the road, but I wasn't hitting something that big with my car!
  - At least three squirrels have tried to commit suicide via my car today
